# High Glucose Aggravates Cerebral Ischemia/Reperfusion via Truncated NLRP3‐Mediated Hexokinase‐2 Translocation

**DOI:** 10.1111/cns.70660

**Published:** 2025-11-18

**Authors:** Hengchang Zhang, Ruoyi Guo, Xiang Li, Yang Zhang, Lujun Zhou, Junjie Wang, Yudi Huang, Zengqiang Yuan, Lijuan Song, Yajin Liao

**Affiliations:** ^1^ Department of Neurology, The Second Affiliated Hospital, Hengyang Medical School University of South China Hengyang China; ^2^ Key Laboratory of Clinical Neurology (Hebei Medical University) Ministry of Education Shijiazhuang Hebei China; ^3^ Department of Anesthesiology, The People's Hospital of Longhua Shenzhen, Affiliated Longhua People's Hospital Southern Medicine University Shenzhen China; ^4^ The Brain Science Center Beijing Institute of Basic Medical Sciences Beijing China; ^5^ The Key Research Laboratory of Benefiting Qi for Acting Blood Circulation Method to Treat Multiple Sclerosis of State Administration of Traditional Chinese Medicine/Research Center of Neurobiology Shanxi University of Chinese Medicine Jinzhong China; ^6^ NHC Key Laboratory of Neurodegenerative Diseases (University of South China) Hengyang China

**Keywords:** high‐glucose, HK2, ischemia/reperfusion, miniNLRP3, NLRP3, PKA

## Abstract

**Background:**

High blood glucose is a well‐established risk factor for poor outcomes in ischemic stroke. However, the underlying molecular mechanisms linking high blood glucose to worsened stroke outcomes remain unclear.

**Objectives:**

Previous studies have implicated the NLRP3 inflammasome, a key mediator of neuroinflammation, in cerebral ischemia/reperfusion (I/R) injury. Under high blood glucose conditions, NLRP3 activation is amplified, potentially driving a vicious cycle of inflammation and neuronal death. Yet, how high blood glucose specifically modulates NLRP3 activation and its downstream pathways remains unclear. This study aimed to investigate the specific mechanisms by which high glucose enhances NLRP3 inflammasome activity and contributes to worsened brain injury following cerebral I/R.

**Methods:**

We employed a combination of in vitro and in vivo experimental approaches to explore the impact of high glucose on NLRP3 inflammasome activation and its consequences on ischemic stroke outcomes. In vitro experiments were conducted by culturing various immune cells in high‐glucose conditions to evaluate the activation of the NLRP3 inflammasome and the mitochondrial association of HK2. In vivo, mice with genetic knockouts of *Nlrp3*, *Pycard* (the gene encoding ASC), or microglial‐specific *Hk2* were subjected to transient middle cerebral artery occlusion (tMCAO).

**Results:**

Our findings revealed that the activation of the NLRP3 inflammasome was enhanced post cerebral I/R under high glucose and a N‐terminal truncation of NLRP3 (miniNLRP3) was induced. Overexpression of PKA could promote the generation of miniNLRP3, while inhibition of PKA decreased the generation of miniNLRP3. In addition, treatment with pan serine protease could block PKA and LPS mediated generation of miniNLRP3. Overexpression of the N‐terminal truncation of NLRP3 could potentiate the activation of the NLRP3 inflammasome under high glucose conditions by promoting the dissociation of Hexokinase 2 (HK2) from mitochondria. In addition, knockout of *Nlrp3*, *Pycard*, or microglial *Hk2*, could all attenuate cerebral I/R‐induced brain injury under high blood glucose in mice.

**Conclusion:**

Our study elucidates PKA‐mediated generation of a 30 kD N‐terminal truncation of NLRP3 (miniNLRP3) in a serine protease‐dependent manner, which could potentiate the activation of the NLRP3 inflammasome under high glucose conditions via promoting the dissociation of HK2 from mitochondria. These findings add a new dimension to our understanding of NLRP3 regulation in the context of stroke injury, and suggest that the PKA‐miniNLRP3‐HK2‐NLRP3 pathway is a promising therapeutic strategy to improve stroke outcomes in patients with elevated blood glucose levels.

## Graphical abstract

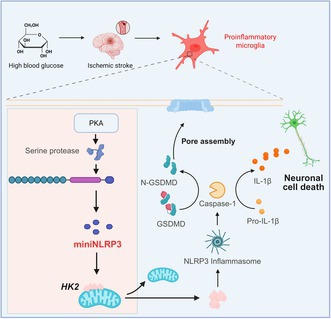

## 1 Introduction

Ischemic stroke (IS) is the major type of stroke, which is caused by thrombus‐induced cerebral ischemia/reperfusion (I/R) and leads to high morbidity and mortality [1]. There are many risk factors that could exacerbate cerebral I/R induced brain injury, such as high blood glucose [2]. Increasing evidence shows that higher blood glucose is positively related to larger infarct volume, higher bleeding risk and poorer prognosis post cerebral I/R [3, 4, 5]. In addition, high blood glucose could counteract the effects of thrombolysis, while blood glucose control has the potential to reduce early stroke mortality [6, 7, 8]. However, the mechanism by which high blood glucose results in deterioration and poor prognosis of patients with ischemic stroke remains obscure.

During the development of IS, many damage‐associated molecular patterns (DAMPs) are released from the dead cells, such as mitochondrial DNA (mtDNA) and ATP [9]. Those DAMPs then activate a robust innate immune response by binding to their receptors, and induce leukocyte infiltration and secondary tissue injury [10, 11]. Taken for example, mtDNA induces the activation of cyclic GMP‐AMP synthase (cGAS), and results in upregulation of pro‐inflammatory factors and more severe tissue injury [9]. Activation of cGAS could further promote pyroptosis via enhancing the activation of NOD‐like receptor protein 3 (NLRP3) and absent in melanoma 2 (AIM2) inflammasome [12, 13]. Apoptosis‐associated speck‐like protein containing a CARD (ASC) is the critical adaptor for both NLRP3 and AIM2 inflammasome formation. The activation of the inflammasome by I/R results in translocation of ASC, cleavage of interleukin (IL‐1β) and gasdermin D (GSDMD) [14, 15]. Then, the cleaved IL‐1β induces a stronger inflammatory response, while the cleaved GSDMD induces pyroptosis of microglia and astrocyte [15, 16]. In addition, more and more evidence shows that the inhibition of cGAS and the inflammasome could reduce the infarct area and improve the outcome of IS [9, 12, 17]. High glucose as a risk factor for IS, is proved to potentiate the activation of the NLRP3 inflammasome in vitro [18, 19]. So, we ask whether high blood glucose deteriorates I/S‐induced brain injury by enhancing the activation of the NLRP3 inflammasome.

Dysfunction of the NLRP3 inflammasome is implicated in plenty of neurodegenerative disorders [20, 21]. Hence, it is important to uncover the mechanism regulating the activation of the NLRP3 inflammasome. In recent years, some endogenous chemicals have been proved to regulate the activation of the NLRP3 inflammasome, such as bile acid and Prostaglandin E2 [22, 23]. Some of the metabolites activate protein kinase A (PKA) by elevating cytosolic cyclic AMP (cAMP) levels; then PKA directly inhibits the activation of NLRP3 by phosphorylating NLRP3 at S295 [22]. However, the activity of PKA is impaired under high glucose due to the decrease of cAMP. So, whether PKA is involved in the upregulation of NLRP3 activity in the development of I/R‐induced brain injury under high glucose is unknown.

Here, we demonstrated that high blood glucose levels aggravate I/R‐induced brain injury via enhancing the activation of the NLRP3 inflammasome. Inhibition of the NLRP3 inflammasome by knockout of *Nlrp3* and *Pycard* (the gene that encodes ASC) could ameliorate I/R‐induced brain injury under high glucose. Interestingly, a N‐terminal truncated NLRP3 was observed in the brain tissue from the high glucose group. The N‐terminal truncated NLRP3 was proved to promote the activation of the NLRP3 inflammasome under high glucose. Mechanically, we found the truncated NLRP3 promoted the mitochondria‐to‐cytoplasm translocation of Hexokinase 2 (HK2), which is essential for the activation of the NLRP3 inflammasome under high glucose. Lastly, knockout of microglial *Hk2* could attenuate I/R‐induced NLRP3 inflammasome activation, brain injury and neurological defects in vivo, thus providing important theoretical evidence for the treatment of high glucose‐induced poor outcomes of IS.

## 2 Materials and Methods

### 2.1 Animal Strains

Hk2 cKO mice (Hk2^flox^) were kindly provided by Professor Jie Zhang from Xiamen University, *Nlrp3* ko and *Pycard* ko mice were kindly provided by Professor Feng Shao from the National Institute of Biological Sciences, Beijing. CX3CR 1^creER‐IRES‐EYFP^ (also named as CX3CR1^creERT2^) transgenic mice (Stock No: 021160) were purchased from the Jackson Laboratory (Sacramento, CA, USA). Male mice were used in the study. All mice were maintained in a specific pathogen‐free animal facility (syxk‐2019‐003) with 12 h light and 12 h dark every day and handled in accordance with the institute's guidelines for the care and use of laboratory animals.

### 2.2 Induction of Hyperglycemia by High‐Fat Diet

To establish a model of hyperglycemia, 8‐week‐old [C57BL/6J] mice were randomly assigned to either a high‐fat diet (HFD) group or a control group. The HFD group was fed a diet containing [e.g., 60% kcal% fat] (D12492, Research Diets, New Brunswick, NJ, USA) for a period of 4 weeks. The control group received a standard normal chow diet (10% kcal% fat). The HFD feeding was continued until the end of the study to maintain the blood sugar levels.

### 2.3 Tamoxifen‐Induced Knockout of *Hk2* in Microglia

Tamoxifen (#S1238, Selleckchem, Houston, TX, USA) was dissolved in sunflower oil and was administered to mice by gavage (dosage: 4 mg/day, 5 consecutive days). According to a previous study, CX3CR1^creERT2^ mediated knock‐out of genes allows for gene manipulation almost exclusively in microglia 4 weeks after tamoxifen administration [24]. So, the experiments were performed 4 weeks after the last time of tamoxifen administration.

### 2.4 Evaluation of Neurologic Deficits

Neurological tests were performed 24 h post‐I/R as previously described [25]. Neurological function was graded on a scale of 0 to 5 as follows: 0 = no deficits; 1 = failure to extend left forepaw fully; 2 = circling to the left; 3 = falling to the left; 4 = no spontaneous walking with a depressed level of consciousness; and 5 = dead.

### 2.5 Western Blotting

Western blot analysis was performed as previously described [9]. The primary antibodies used in the present study were anti‐NLRP3 (#AG‐20B‐0014, AdipoGen, San Diego, CA, USA, 1:1000 for Western blotting, 2 μg/sample for immunoprecipitation), anti‐Casp‐1 (#Ag‐20B‐0042, AdipoGen, 1:1000), anti‐ASC (#67824, Cell Signaling Technology, Beverly, MA, USA, 1:1000 for Western blotting, 1:200 for immunofluorescence), anti‐IL1β (#AF‐401‐NA; R&D Systems, 1:1000), anti‐Hexokinase (#EPR20839, Abcam, 1:1000 for Western blotting, 1:200 for immunofluorescence), anti‐TOM20 (#42406, Cell Signaling Technology, 1:1000 for Western blotting), anti‐GSDMD (#ab219800, Abcam, 1:1000 for Western blotting, 1:200 for immunofluorescence), anti‐β‐actin (#P01L082, Gene‐Protein Link, 1:1000 for Western blotting), anti‐Flag tag (#F3165, Sigma‐Aldrich, 1:2000 for Western blotting), anti‐Iba1 (#N100‐1028, Novus Biologicals, 1:200 for immunofluorescence), anti‐Iba1 (#17198, Cell Signaling Technology, 1:1000 for Western blotting), anti‐HA tag (#AE008, Abclonal, 1:1000 for Western blotting), anti‐Myc tag (#E022050‐01, EARTH, 1:1000 for Western blotting), anti‐β‐tubulin (#CW0265, CWBIO, 1:1000 for Western blotting), anti‐PKA (#4782, CST, 1:1000 for Western blotting).

### 2.6 Cell Lines and Culture

BV2 microglial cells, immortalized bone marrow‐derived macrophages (iBMDMs) and HEK293T cells were maintained in Dulbecco's modified Eagle's medium (DMEM) (#11965–092, Life Technologies) supplemented with 10% heat‐inactivated fetal bovine serum (FBS, #04–001‐1A, Biological Industries, Beit Haemek, Israel) and 1% penicillin–streptomycin solution (#03–031‐1B, Biological Industries) at 37°C in a humidified atmosphere with 5% CO_2_. Lentiviral transfer vectors pSIN‐miniNLRP3 were created by cloning miniNLRP3 into the pSin‐GFP‐Fg vector. iBMDM cell lines stably expressing miniNLRP3 were constructed by lentivirus‐mediated transfection and screened with puromycin.

### 2.7 Activation of the NLRP3 Inflammasome

Plate the appropriate cells in cell culture plates overnight in complete growth medium under standard conditions (37°C, 5% CO_2_) to allow them to adhere and reach ~80% confluency. The following day, aspirate the growth medium and replace it with fresh medium containing a sublytic concentration of Ultra‐Pure Lipopolysaccharide (LPS) (1 μg/mL). Incubate the cells for approximately 4 h. This “priming” step is critical for upregulating the expression of NLRP3 and pro‐IL‐1β via the TLR4 signaling pathway, preparing the cells for inflammasome activation.

For the ATP stimulation group: Gently add fresh, pre‐warmed medium containing a specific concentration of ATP (5 mM). Incubate the cells for a short duration (30 min to 1 h). ATP induces a rapid K^+^ efflux through the P2X7 receptor, a canonical trigger for NLRP3 inflammasome assembly.

For the nigericin stimulation group: Gently add fresh, pre‐warmed medium containing a specific concentration of nigericin (6.7 μM). Incubate the cells for a shorter duration (e.g., 30 min to 1 h). Nigericin acts as a K^+^/H^+^ ionophore, potently inducing K^+^ efflux to activate NLRP3.

### 2.8 Real‐Time Quantitative PCR

RNA was extracted from tissue and cells using Trizol reagent (#15596026, Life Technologies) according to the manufacturer's instructions. Then, mRNA was converted to cDNA using a StarScript Pro All‐in‐one RT Mix with gDNA Remover kit (A240, GenStar) according to the manufacturer's protocol. Finally, the cDNA was used for real‐time quantitative PCR (qPCR) with 2 × RealStar Fast SYBR qPCR Mix (#A304, GenStar) and STUDIO Q3 (ABI, Life Technologies).

### 2.9 Transient Middle Cerebral Artery Occlusion (tMCAO) Model

Male Mice weighing approximately 22 g were anesthetized with an intraperitoneal injection of pentobarbital (0.7%, 10 μL/g body weight). To perform tMCAO, a silicon‐coated nylon suture (#602156PK5Re, Doccol, Sharon, MA, USA) was advanced into the MCA for 60 min and the blood flow was monitored by laser Doppler velocimetry (PF5001, PERIMED, Stockholm, Sweden). Male Mice that underwent sham surgery (without blocking of the blood flow by nylon suture) were used as controls. For a high glucose diet, the mice were fed with a high dextrose diet (#PD2311101, SYSE), which contains 57.8 g% of dextrose.

### 2.10 Infarct Volume Measurements

The mice were anesthetized and sacrificed 24 h post‐tMCAO. The brains were taken out and cut into 1 mm thick slices, and incubated in a 1.5% 2,3,5‐triphenyl tetrazolium chloride (TTC) solution for 5 min at 37°C. The infarct volumes of each slice were determined using ImageJ and summed to determine the percentage of whole infarct volume. As the infarct volumes were calculated by the total TTC negative area verses the total brain slice area $\left(\text{Infarct volume}=\sum_{n=1}^{8}\left(\mathrm{TTC}\;\text{negative area}\right)/\sum_{n=1}^{8}\left(\text{brain slice area}\right)\times 100\%\right)$, so, the edema was considered during the calculation of infarct volume as well.

### 2.11 Purification of Adult Microglia

The mice brain was first dissociated into single‐cell suspension according to the instructions of the Adult Brain Dissociation Kit (#130–107‐677, Miltenyi) with gentleMACS equipment (Miltenyi); then microglia were purified with CD11b MicroBeads (#130–097‐142, Miltenyi) according to the manual's instructions with the MS Columns (#130–042‐201, Miltenyi).

### 2.12 Isolation and Culture of Newborn Primary Microglia

Newborn mice were sacrificed and the cortices were taken out, followed by digesting with 0.25% trypsin at 37°C for 10 min. Then, an equal volume of 10% FBS/DMEM plus DNase I (100 ng/mL) was added and incubated at 37°C for 1 min. The suspension was pipetted into a single cell suspension and centrifuged at 250 ***g*** for 5 min. The pellet was re‐suspended in 10% FBS/DMEM plus recombinant mouse nerve growth factor (5 ng/mL, #784004, BioLegend) and recombinant murine fibroblast growth factor‐basic (10 ng/mL, #AF‐450‐33, PeproTech). Then, the single cell suspension was transferred to a poly L‐ornithine‐coated T‐25 flask and cultured in an incubator (37°C, 5% CO_2_). The medium was replaced with fresh medium every other day. Eight days later, the microglia were purified by shaking the flask at a speed of 200 rpm for 5 min, and the cells in the medium were harvested by centrifuging. Cells were resuspended in 1 mL fresh medium and were counted with an automated cell counter (Countess TM II FL, Life Technologies) for seeding. The purity of microglia was confirmed by immunostaining with anti‐Iba1 antibody.

### 2.13 Immunostaining and Imaging

Slices or fixed cultured cells were permeabilized with 0.5% Triton X‐100 and blocked with PBS plus 10% horse serum, followed by incubating with primary antibodies overnight at 4°C. Then, the samples were washed with PBS with Tween 20 (PBST) 4 times and incubated with fluorescently labeled secondary antibodies at room temperature for 90 min. Finally, the samples were washed once with PBST, the nuclei were stained with 4′,6‐diamidino‐2‐phenylindole (DAPI, #D9542, Sigma‐Aldrich), and the samples were washed with PBST a further 3 times. Then, the slices were mounted with Antifade Mountant (#P36961, ThermoFisher) and coverslips. Images were captured with a laser confocal microscope (Ti‐A1, NIKON, Minato‐ku, Tokyo, Japan) and slide scanner (Pannoramic SCAN, 3DHISTECH, Budapest, Hungary).

### 2.14 Cloning and Plasmid Construction

The open‐reading frame (ORF) of human PKA was cloned into pCDNA3.1‐HA vector by polymerase chain reaction (PCR). The full‐length or truncated variants ORF of human NLRP3 were cloned into pCMV10‐3 × Flag Vector. The mutants that code for PKA^K72H^, NLRP3^R262W^, or NLRP3^S295A^ were constructed by PCR with designed mutation primers (Table 1) and the plasmids encoding the wild‐type gene, respectively. For the establishment of the NLRP3_1‐262_ overexpression cell line, the gene encoding the N‐terminal 1‐262aa of NLRP3 was cloned into pSin‐GFP‐Fg vector.

**TABLE 1:** The primers used in this study (5′‐3′).

Primers	Sequence (5′‐3′)
*Pkaα* ^K72H^‐F	CCACTACGCCATGCACATCTTAGACAAGCAGAAGGTGGTG
*Pkaα* ^K72H^‐R	TCTGCTTGTCTAAGATGTGCATGGCGTAGTGGTTCCCACTC
*Nlrp3* ^R262W^‐F	TGTTCTATATCCACTGTTGGGAGGTGAGCCTTGTGACAC
*Nlrp3* ^R262W^‐R	GGCTCACCTCCCAACAGTGGATATAGAACAGATAGTCAA
*Nlrp3* ^S295A^‐F	CGTGAGAAAACCCGCCAGAATCCTCTTCCTCATGGACGG
*Nlrp3* ^S295A^‐R	GAAGAGGATTCTGGCGGGTTTTCTCACGATCTTGTGGATG
*Nlrp3* ^1‐262^‐F	GTTCCGCGTGGATCCCCGGAAATGAAGATGGCAAGCACCCGCTG
*Nlrp3* ^1‐262^‐R	CGCTCGAGTCGACCCGGGCTACCGACAGTGGATATAGAACAG
*Nlrp3* ^1‐282^‐F	GATCTCGAGCTCAAGCTTCGATGGATTACAAAGACGATGACG
*Nlrp3* ^1‐282^‐R	CGCGGTACCGTCGACTGCAGCTAGTCGGGGCAGCAGCTCATG
*Nlrp3* ^1‐337^‐F	GATCTCGAGCTCAAGCTTCGTGGATTACAAAGACGATGACG
*Nlrp3* ^1‐337^‐R	CGCGGTACCGTCGACTGCAGCTATCTGATGAGGCTGCTCAGG
*Nlrp3* ^Δ233–252^‐F	GGGATTGGGAAA TTTGACTATCTGTTCTATATCCACTGTC
*Nlrp3* ^Δ233–252^‐R	ATAGAACAGATAGTCAAA TTTCCCAATCCCTGCCGCCCCC
*Nlrp3* ^Δ263–282^‐F	ATATCCACTGTCGG CCAAACCCACCCATCCACAAGATCG
*Nlrp3* ^Δ263–282^‐R	GGGTGGGTTTGG CCGACAGTGGATATAGAACAGATAGTC
*Nlrp3* ^Δ253–292^‐F	CTCTACCAAGACAGG AAACCCTCCAGAATCCTCTTCCTC
*Nlrp3* ^Δ253–292^‐R	TTCTGGAGGGTTT CCTGTCTTGGTAGAGTGTCCCCGA
*Nlrp3* ^Δ293–312^‐F	CACAAGATCGTGAGAGAGCACATAGGACCGCTCTGCACTG
*Nlrp3* ^Δ293–312^‐R	GGTCCTATGTGCTC TCTCACGATCTTGTGGATGGGTGGG
*Nlrp3* ^Δ313–332^‐F	AGGTGCCTTTGAC AGCAGCCTCATCAGAAAGAAGCTGCT
*Nlrp3* ^Δ313–332^‐R	TGATGAGGCTGCT GTCAAAGGCACCTTGCAGCTCATCGA
*Nlrp3*‐ko	AAGTCGTGCTGCTTCATGT
*Nlrp3*‐com	TCAAGCTAAGAGAACTTTCTG
*Nlrp3*‐wt	ACACTCGTCATCTTCAGCA
sm99‐gASC	CTAGTTTGCTGGGGAAAGAAC
sm105‐sASC	CTAAGCACAGTCATTGTGAGCTCC
sm141‐neoASC	AAGACAATAGCAGGCATGCTGG

### 2.15 Enzyme‐Linked Immunosorbent Assay (ELISA)

The culture medium was collected after cell stimulation and then centrifuged at 3000 ***g*** for 5 min. The concentration of IL‐1β in the supernatants was determined by ELISA according to the manufacturer's instructions (#432604, BioLegend, San Diego, CA, USA).

### 2.16 ASC Oligomerization Assay

iBMDM cell lines were exposed to 1 μg/mL LPS for 5 h, and then stimulated with ATP (5 mM) for 1 h. The supernatant was removed, and the cells were rinsed in ice‐cold PBS and then lysed with hypotonic lysis buffer (10 mM KCl, 1.5 mM MgCl_2_, 1 mM EDTA, 1 mM EGTA, 0.1 mM PMSF, and 20 mM Tris; pH 7.5) for 30 min at 4°C. The lysates were centrifuged at 6000 ***g*** for 8 min at 4°C, and the supernatants contained the soluble components. The pellets were washed twice in 1 mL ice‐cold PBS and resuspended in 500 μL CHAPS buffer (0.1% CHAPS, 10 mM KCl, 1.5 mM MgCl_2_, 1 mM EDTA, 1 mM EGTA, 0.1 mM PMSF, and 20 mM Tris; pH 7.5). Disuccinimidyl suberate (DSS, #S1885, Sigma‐Aldrich, 2 mM) was added to the resuspended pellets, which were incubated at 37°C for 45 min with rotation. The samples were then centrifuged at 6000 ***g*** for 15 min. The crosslinked pellets were resuspended in 60 μL SDS loading buffer and were heat denaturized. Then, oligomeric ASC levels were analyzed by Western blotting.

### 2.17 Isolation of Mitochondria

Isolation of cytosolic and mitochondrial fraction was conducted by using a Mitochondria Isolation Kit (#C3601, Beyotime Biotechnology) for Cultured Cells following the manufacturer's protocol.

### 2.18 NeuN and TUNEL Co‐Staining

The tissue slices were placed in 2 mL 0.1 M citric acid buffer (pH 6.0) and repaired at 95°C for 35 min. Then, the slices were washed with PBS three times for 5 min. Subsequently, the TUNEL reaction mixture was added to the slice and incubated for 60 min at 37°C in the dark. Followed by washing with PBS three times. Then, the slices were blocked with 5% FBS/PBS plus 0.3% Triton X‐100, and then incubated with the NeuN primary antibody at 4°C overnight. On the second day, after incubating with the TRITC‐labeled secondary antibody, the nucleus was stained with DAPI. The image was captured by laser confocal microscopy (Ti‐A1, Nikon, Tokyo, Japan).

### 2.19 siRNA Transfection

Primary microglia cells were then transfected with 50 nM nontargeting siRNA or siRNA against HK2 using Lipofectamine RNAi MAX transfection reagent (#13778075, Invitrogen) following the manufacturer's protocol; the cells were used for further study at 72 h post transfection (hpt).

### 2.20 Behavior Test

Tail suspension test. Mice were suspended by the tail on a station (50 cm above the floor) for 6 min and recorded by camera. The latency to the first period of immobility during the test and the cumulative immobility time during the final 4 min were analyzed by Smart V3.0 (Panlab, Spain).

Forced swimming test. This test was performed in a clear glass cylinder (height 45 cm, diameter 19 cm), which was filled with 2 L of water (18°C). The duration of the 6‐min test was recorded by a camera, and the cumulative immobility time within the final 4 min was analyzed by Smart V3.0.

### 2.21 Blinding and Randomization

All experimental animals were randomly assigned to different treatment groups using a random number generator. For all surgical procedures, outcome assessments, and data analysis, the investigators were blinded to the group allocation. Specifically, the researchers performing the cerebral infarct volume measurements (via TTC staining) and conducting the behavioral tests (e.g., neurological deficit scoring, adhesive removal test, etc.) were different from the one performing the surgeries and treatments and were provided with coded samples and animals whose group identity was concealed until all analyses were completed.

### 2.22 Statistical Analyses

All experimental data are presented as mean ± Standard Error of the Mean (SEM) and were analyzed using GraphPad Prism version 10 (GraphPad Software, San Diego, CA, USA). Normality of data distribution was assessed using the Shapiro–Wilk test. For data that followed a normal distribution, differences between two groups were evaluated using Student's *t*‐test, while comparisons among multiple groups were conducted using one‐way analysis of variance (ANOVA) followed by Bonferroni's post hoc test. For data that did not meet the assumption of normality, non‐parametric equivalents were employed: the Mann–Whitney *U* test for two‐group comparisons and the Kruskal–Wallis test followed by Dunn's post hoc test for multiple groups. Differences in behavioral scores across groups were analyzed using two‐way repeated measures ANOVA with Bonferroni correction. For all statistical analyses, *p* < 0.05 was interpreted as statistically significant.

## 3 Results

### 3.1 High Glucose Enhances the Cerebral I/R Induced Activation of NLRP3 Inflammasome

**FIGURE 1:**
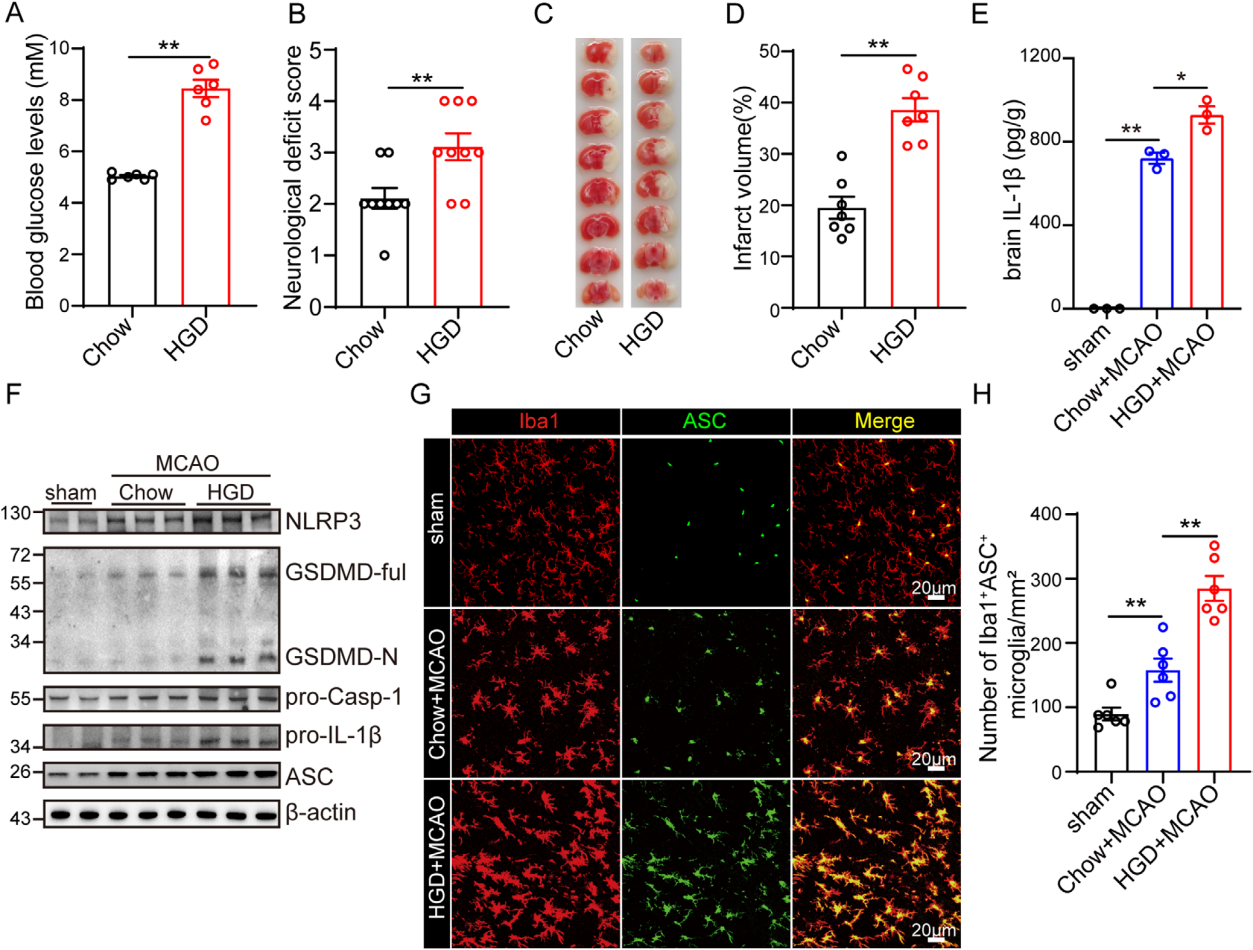
High‐glucose diet (HGD) aggravates cerebral ischemia/reperfusion (I/R) induced brain injury and NLRP3 inflammasome activation. (A) The blood glucose levels in mice fed with chow (*n* = 6) or HGD (*n* = 6) were determined by glucose meter. (B) The neurological deficits of mice fed with chow (*n* = 9) or HGD (*n* = 9) were assessed 24 h post‐reperfusion. (C, D) Brain tissue isolated from mice fed with chow (*n* = 7) or HGD (*n* = 7) 24 h post‐reperfusion was stained with triphenyl tetrazolium chloride (TTC), and infarct volume was determined using ImageJ. (E) The concentration of interleukin‐1β (IL‐1β) in the brain of mice fed with chow (*n* = 3) or HGD (*n* = 3) 24 h post‐reperfusion and sham operation was determined by Enzyme‐Linked Immunosorbent Assay (ELISA). (F) The protein levels of NLRP3, GSDMD, pro‐Casp 1, Pro‐IL‐1β, ASC, and β‐Actin in the ischemic penumbra brain tissue from mice fed with chow or HGD 24 h post‐reperfusion and sham operation were detected by western blot. (G, H) The number of ASC^+^Iba1^+^ cells in the ischemic penumbra cortex of mice fed with chow or HGD subjected to tMCAO 24 h post‐reperfusion and sham operation was detected by indirect immunofluorescent staining and captured by co‐focal microscope. (* indicates *p* < 0.05, ** indicates *p* < 0.01 by ANOVA or Student's *t*‐test).

To prove that high blood glucose would lead to poor outcomes of cerebral I/R induced brain injury, we firstly performed the tMCAO with mice fed with Chow and high‐glucose diet (HGD). The mice fed with HGD displayed higher blood glucose levels and increased neurological deficit scores (Figure 1A,B). In addition, the infarct volume 24 h post‐I/R was significantly increased in the HGD group as well (Figure 1C,D). Consistently, cerebral I/R induced expression of IL‐1β in the brain was further potentiated by HGD (Figure 1E). As a result, we further determined the activation of the NLRP3 inflammasome in the brain tissue that underwent I/R. The protein levels of NLRP3, ASC, cleaved‐Caspase‐1, Caspase‐1, cleaved‐IL‐1β, and IL‐1β were used to evaluate the activation of the NLRP3 inflammasome. We found that the activation of the NLRP3 inflammasome was intensified after I/R injury in mice fed with HGD (Figure 1F). What's more, the protein levels of cleaved GSDMD were also further upregulated by HGD (Figure 1F). Additionally, HGD significantly aggravates microglia activation and increases the number of ASC positive microglia (Figure 1G,H).

### 3.2 Inhibition of NLRP3 Inflammasome Could Attenuate I/R‐Induced Brain Injury Under HGD

**FIGURE 2:**
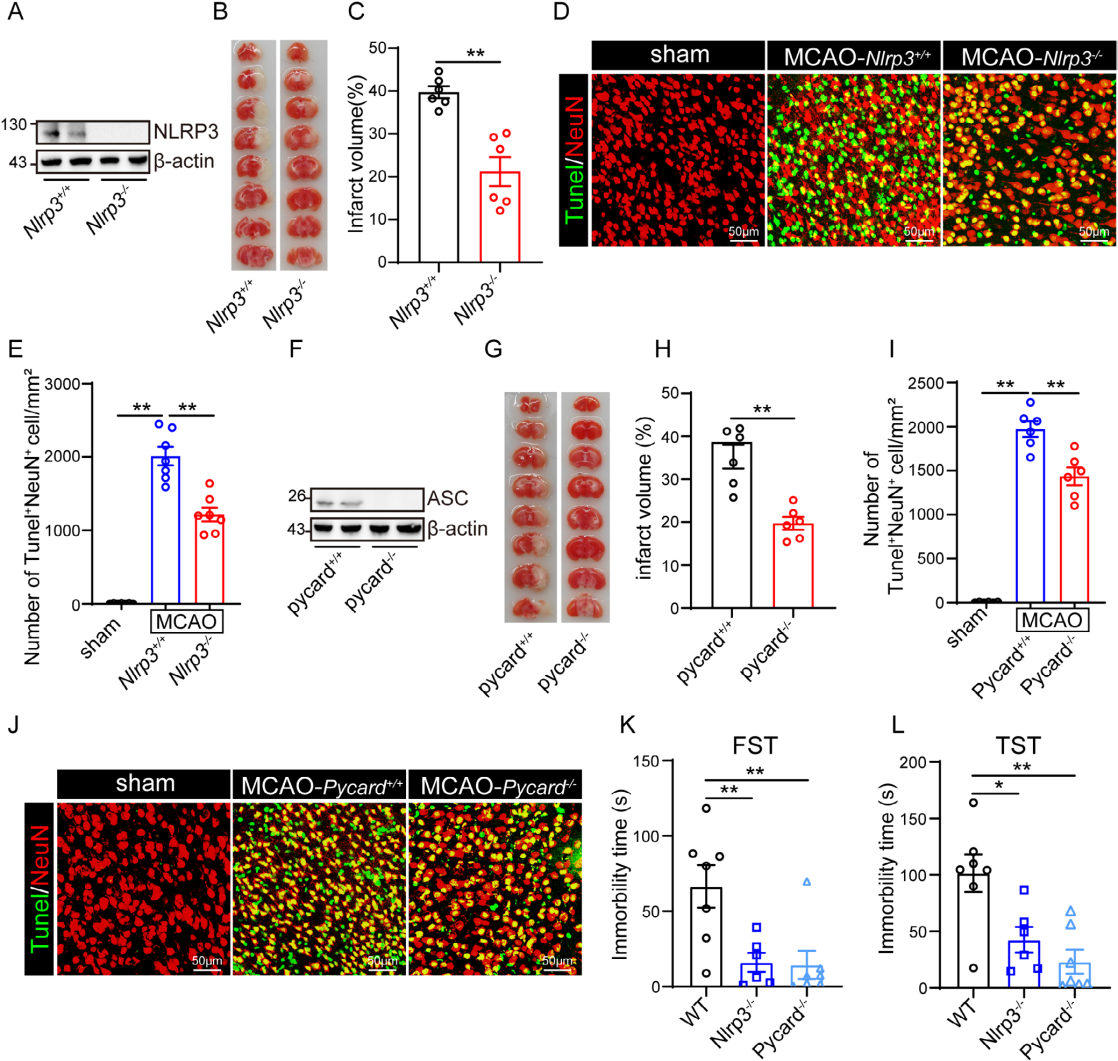
Knockout of *Nlrp3* and *Pycard* could attenuate cerebral I/R‐induced brain injury fed with HGD. (A) The protein level of NLRP3 and β‐Actin in the cortex of *Nlrp3* ^+/+^ and *Nlrp3* ^−/−^ mice were detected by western blot. (B, C) Brain tissue isolated from *Nlrp3* ^+/+^ (*n* = 6) and *Nlrp3* ^−/−^ (*n* = 6) mice fed with HGD 24 h post‐reperfusion was stained with TTC, and infarct volume was determined using ImageJ. (D, E) The number of TUNEL^+^NeuN^+^ cells in the ischemic penumbra cortex of *Nlrp3* ^+/+^ (*n* = 6) and *Nlrp3* ^−/−^ (*n* = 6) mice fed with HGD 24 h post‐reperfusion was stained with TUNEL kit and indirect immunofluorescent staining, and the pictures were captured by confocal microscope. (F) The protein level of ASC and β‐Actin in the cortex of *Pycard* ^+/+^ and *Pycard* ^−/−^ mice were detected by western blot. (G‐H) Brain tissue isolated from *Pycard* ^+/+^ (*n* = 6) and *Pycard* ^−/−^ (*n* = 6) mice fed with HGD 24 h post‐reperfusion was stained with TTC, and infarct volume was determined using ImageJ. (I, J) The number of TUNEL^+^NeuN^+^ cells in the ischemic penumbra cortex of *Pycard* ^+/+^ (*n* = 6) and *Pycard* ^*−/−*^ (*n* = 6) mice fed with HGD 24 h post‐reperfusion was stained with TUNEL kit and indirect immunofluorescent staining, and the pictures were captured by confocal microscope. (K, L) WT (*n* = 7), *Nlrp3* ^−/−^ (*n* = 6), and Pycard^−/−^ (*n* = 6) *Pycard* ^−/−^ (*n* = 7) mice underwent MCAO/R were fed with HGD for 14d, then the depression‐like behaviors were tested by forced swimming test (K) and tail suspension test (L) (* indicates *p* < 0.05, ** indicates *p* < 0.01 by ANOVA or Student's *t*‐test).

The above results indicated that high blood glucose levels exacerbated I/R‐induced brain injury and potentiated the activation of the NLRP3 inflammasome. So, we asked whether inhibition of the NLRP3 inflammasome could ameliorate I/R‐induced brain injury under HGD. Firstly, *Nlrp3* knockout mice were used for the study (Figure 2A). As the results show, the infarct volume was decreased in the brain of the *Nlrp3* ko mice (Figure 2B,C). In addition, the number of TUNEL positive neurons was also reduced by the knockout of *Nlrp3* (Figure 2D,E). Due to ASC being the critical adaptor for NLRP3 inflammasome formation, and the loss of ASC would abolish the activation of the NLRP3 inflammasome, *Pycard* knockout mice were used for further confirmation (Figure 2F). Consistently, the infarct volume and the number of TUNEL positive neurons were both decreased in the *Pycard* knockout mice (Figure 2G–J). In addition, the FST (Figure 2K) and TST (Figure 2L) displayed that the knockout of *Nlrp3* and *Pycard* ameliorated the depression‐like behaviors at 14d post I/R.

### 3.3 A N‐Terminal Truncated NLRP3 Was Observed In Vivo and In Vitro Upon the Activation of NLRP3 Inflammasome

**FIGURE 3:**
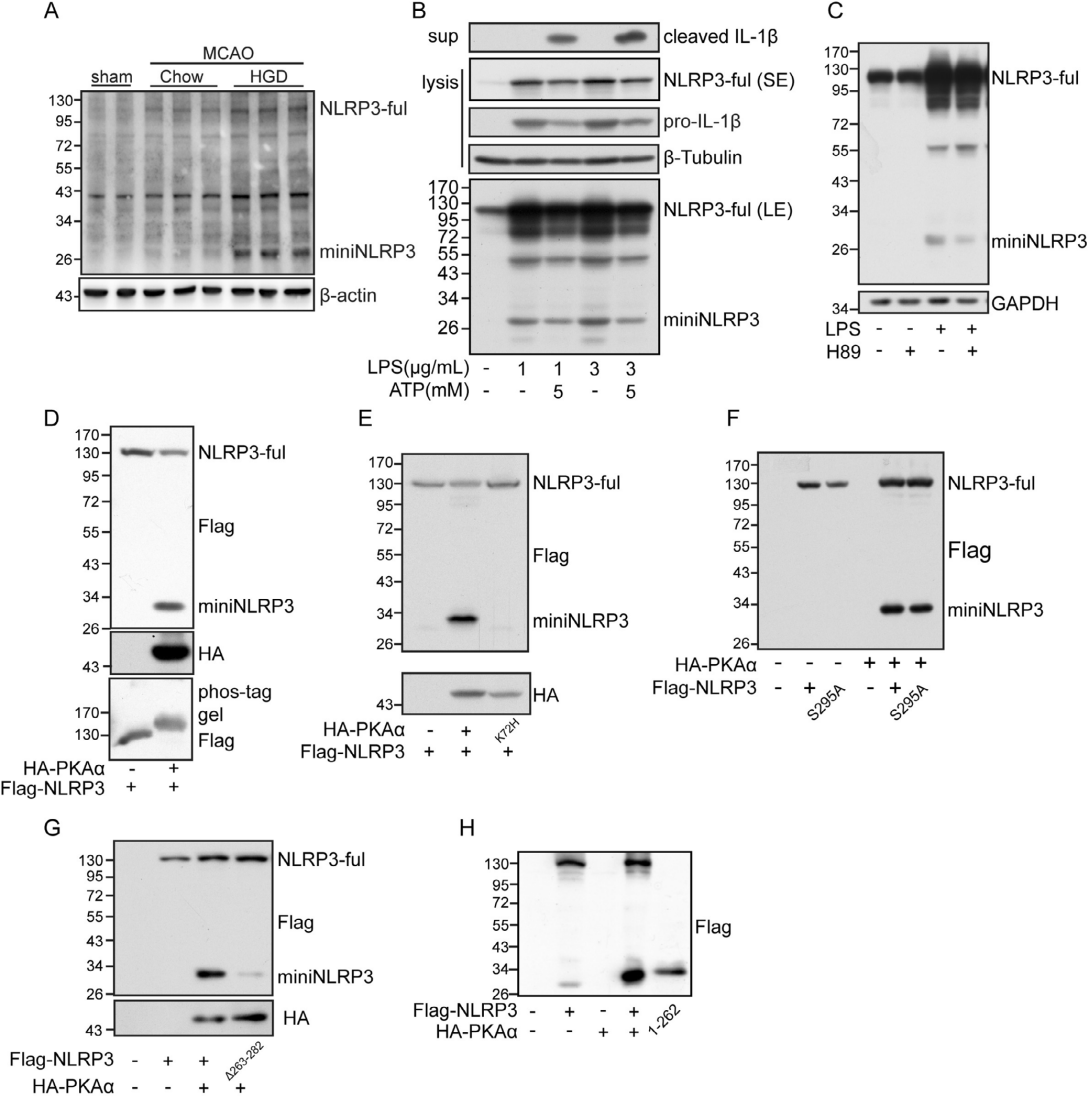
A N‐terminal truncated NLRP3 was observed in vivo and in vitro upon the activation of the NLRP3 inflammasome. (A) The protein levels of full‐length NLRP3, miniNLRP3 and β‐Actin in the ischemic penumbra brain tissue from mice fed with chow or HGD 24 h post‐reperfusion and sham operation were detected by western blot. (B) iBMDM cells exposed to lipopolysaccharide (LPS) for 4 h were followed by stimulation with ATP for another 1 h; then, the protein levels of full‐length NLRP3, miniNLRP3, Pro‐IL‐1β, and β‐tubulin in cells and cleaved IL‐1β in the supernatant were detected by western blot. (C) iBMDM cells pretreated with H89 (20 μM) for 30 min were exposed to LPS (1 μg/mL) for 4 h, and the protein levels of full‐length NLRP3, miniNLRP3 and GAPDH were detected by western blot. (D) HEK293T cells transfected with pFlag‐NLRP3 or pFlag‐NLRP3 plus pHA‐PKA were collected 24 h post‐transfection, and the protein levels of full‐length NLRP3, miniNLRP3 and HA‐PKA were detected by western blot, while the phosphorylation levels of NLRP3 were detected by phos‐tag gel and western blot. (E) HEK293T cells transfected with pFlag‐NLRP3, pFlag‐NLRP3 plus pHA‐PKA or pFlag‐NLRP3 plus pHA‐PKA K72H mutant were collected 24 h post‐transfection (hpt), and the protein levels of full‐length NLRP3, miniNLRP3 and HA‐PKA were detected by western blot. (F) HEK293T cells transfected with pFlag‐NLRP3, pFlag‐NLRP3 plus pHA‐PKA or pFlag‐NLRP3 S295A mutant plus pHA‐PKA were collected 24 hpt, and the protein levels of full‐length NLRP3, miniNLRP3 and HA‐PKA were detected by western blot. (G) HEK293T cells transfected with pFlag‐NLRP3, pFlag‐NLRP3 plus pHA‐PKA or pFlag‐NLRP3 Δ263‐282 mutant plus pHA‐PKA were collected 24 hpt, and the protein levels of full‐length NLRP3, miniNLRP3 and HA‐PKA were detected by western blot. (H) HEK293T cells transfected with pFlag‐NLRP3, pFlag‐NLRP3 plus pHA‐PKA, pFlag‐NLRP3_1‐262_ truncation, or pFlag‐NLRP3_1‐282_ truncation were collected 24 hpt, and the protein levels of full‐length NLRP3, miniNLRP3, and HA‐PKA were detected by western blot.

We next investigated the underlying mechanism that HGD enhances the activation of the NLRP3 inflammasome in cerebral I/R. Interestingly, a 30 kD band was recognized by an antibody against the N‐terminal of NLRP3 in the brain tissue from HGD mice that underwent cerebral I/R, which was named miniNLRP3 in the current study (Figure 3A). In addition, miniNLRP3 was also observed in the cell lysis stimulated with LPS (Figure 3B), and the production of miniNLRP3 was inhibited by H89 (20 μM) (Figure 3C), the inhibitor of protein kinase A (PKA). Furthermore, overexpression of PKA resulted in a similar band in vitro, and upregulated the phosphorylation levels of NLRP3 (Figure 3D). While the kinase‐dead PKA mutant failed to mediate the production of miniNLRP3 (Figure 3E). These results indicate the production of miniNLRP3 is dependent on the kinase activity of PKA. The S295 site was reported to be phosphorylated by PKA to promote the degradation of NLRP3 under the activation of the NLRP3 inflammasome. However, the substitution of serine with alanine at S295 and has no effect on PKA‐mediated production of miniNLRP3 (Figure 3F). To uncover whether PKA directly mediates the generation of miniNLRP3, several protease inhibitors were used. The results indicated that inhibition of serine protease with AEBSF could block the generation of PKA and LPS‐induced generation of miniNLRP3 (Figure S1A,B). However, pretreatment with caspase inhibitors has no significant effect on PKA‐mediated generation of miniNLRP3 (Figure S1C). To further study the role of miniNLRP3, a series of NLRP3 mutants was constructed and co‐transfected with PKA, and only the mutant missing the 263–282aa significantly decreased the production of miniNLRP3 (Figure 3G and Figure S2). So, we constructed the N‐terminal 1–262 aa and 1–282 aa NLRP3 mutants, and the 1–262 aa has the same size as miniNLRP3 (Figure 3H).

### 3.4 NLRP3_1_ _‐262_ Truncation Promotes the Activation of NLRP3 Inflammasome Under High Glucose

**FIGURE 4:**
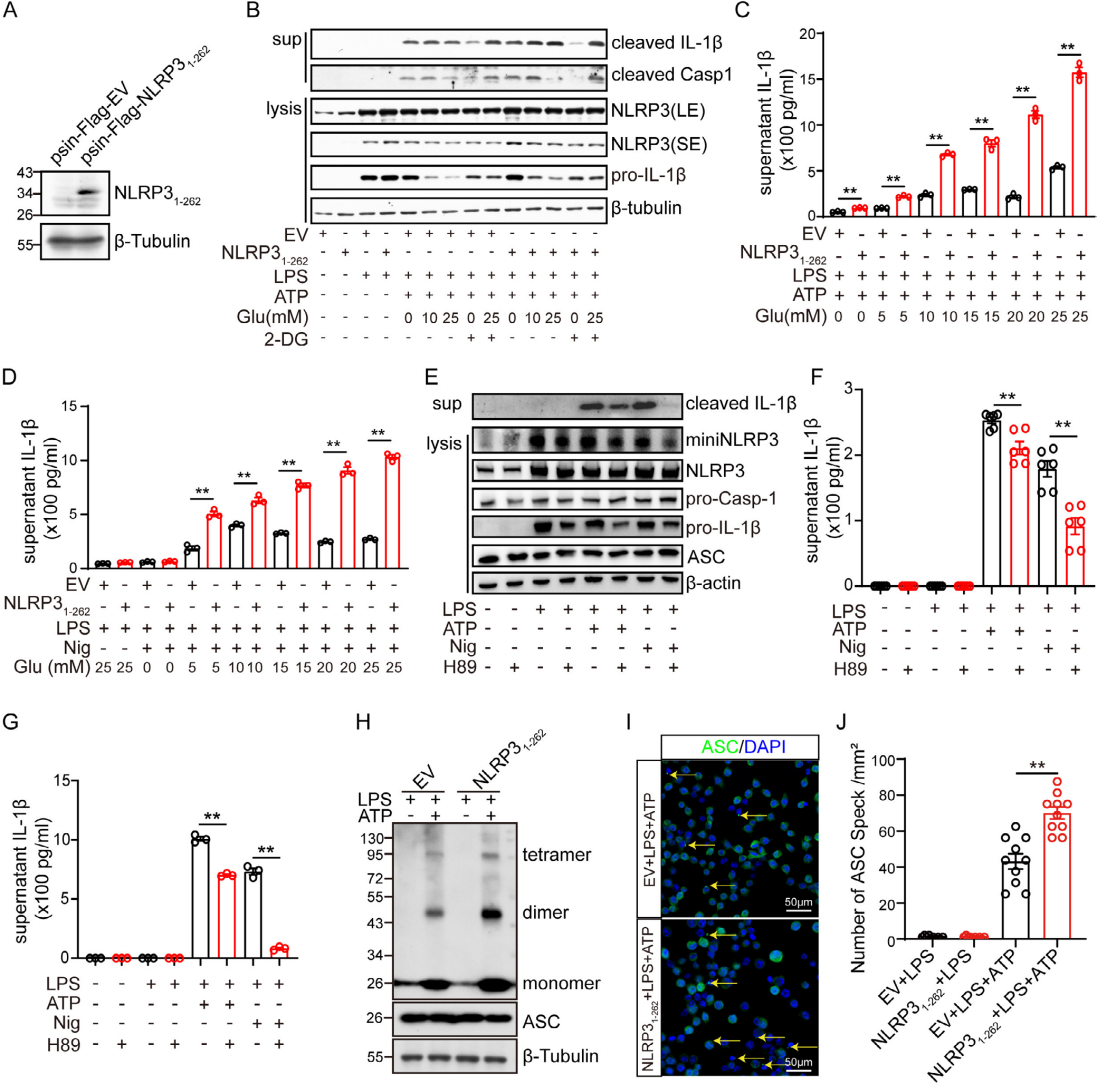
NLRP3_1‐262_ truncation promotes the activation of NLRP3 inflammasome under high glucose. (A) The protein levels of NLRP3_1‐262_ and β‐tubulin in iBMDM cells that stably express empty vector or Flag‐NLRP3_1‐262_ were detected by western blot. (B) iBMDM cells that stably express empty vector or Flag‐NLRP3_1‐262_ were cultured in medium with 0, 10, and 25 mM glucose (Glu) and 2‐Deoxy‐D‐glucose (2‐DG) were exposed to LPS (1 μg/mL) for 4 h, followed by stimulation with ATP (5 mM) for another 45 min. Then the protein levels of cleaved IL‐1β, cleaved Casp 1, NLRP3, pro‐IL‐1β and β‐tubulin in cell lysis and medium were detected by western blot. (C, D) iBMDM cells that stably express EmptyVector (EV) or Flag‐NLRP3_1‐262_ were cultured in medium with 0, 5, 10, 15, 20, and 25 mM glucose 30 min before exposure to LPS (1 μg/mL) for 4 h, followed by stimulation with ATP (5 mM) (C) or Nig (6.7 μM) (D) for another 45 min, then the concentration of IL‐1β in the supernatant was determined by ELISA. (E‐F) iBMDM pretreated with or without H89 (20 μM) for 30 min were exposed to LPS for 4 h, then the medium was replaced with fresh medium and cells were further stimulated with ATP (5 mM) or Nig (6.7 μM) for another 45 min. The protein levels of full‐length NLRP3, miniNLRP3, pro‐IL‐1β, pro‐Casp 1, ASC and β‐tubulin in cells and cleaved‐IL‐1β in the supernatant were detected by western blot (E) and the supernatant IL‐1β concentration was determined by ELISA (F). (G) Primary microglia pretreated with or without H89 (20 μM) for 30 min were exposed to LPS for 4 h, then the medium was replaced with fresh medium and cells were further stimulated with ATP (5 mM) or Nig (6.7 μM) for another 30 min, then the supernatant IL‐1β concentration was determined by ELISA. (H) iBMDM cells that stably express empty vector or Flag‐NLRP3_1‐262_ were cultured in medium with 25 mM glucose were exposed to LPS (1 μg/mL), followed by stimulation with ATP (5 mM), then the cells were harvested and crosslinked with disuccinimidyl suberate for analysis of the oligomerization of ASC by western blot. (I, J) iBMDM cells that stably express empty vector or Flag‐NLRP3_1‐262_ were cultured in medium with 25 mM glucose were exposed to LPS (1 μg/mL), followed by stimulation with ATP (5 mM), then the cells were fixed and stained with anti‐ASC antibody and the number of ASC specks was analyzed. (* indicates *p* < 0.05, ** indicates *p* < 0.01 by ANOVA or Student's *t*‐test).

The above results indicate that miniNLRP3 might be the N‐terminal 1‐262aa of NLRP3, so we constructed an iBMDM cell line that stably expresses the N‐terminal 1‐262aa of NLRP3 for its function study (Figure 4A). The cells expressing NLRP3_1‐262_ or empty‐vector (EV) were exposed to LPS (1 μg/mL) in the medium including 0‐, 10‐, and 25‐mM glucose, and followed by stimulating with ATP (5 mM) or Nig (6.7 μM). The results displayed that overexpression of NLRP3_1‐262_ significantly increased the expression of cleaved IL‐1β at 10‐ and 25‐mM glucose when stimulated with ATP (Figure 4B). The ELISA results indicated that the concentration of IL‐1β in the supernatant from the NLRP3_1‐262_ overexpressed iBMDM was increased with the increasing glucose; however, the concentration of IL‐1β in the supernatant from the EmptyVector overexpressed iBMDM was not significantly upregulated when the concentration of glucose was higher than 10 mM (Figure 4C,D). As the above results displayed that H89 could inhibit the production of miniNLRP3, we then determined the activation levels of the NLRP3 inflammasome in cells pre‐treated with H89 (20 μM) 30 min before adding LPS. The results showed that cleaved IL‐1β and the concentration of IL‐1β in the supernatants from iBMDM (Figure 4E,F) and primary microglia (Figure 4G) were both downregulated by H89 under high glucose. However, pre‐treatment of cells with H89 30 min before adding ATP or Nig increased the amount of cleaved IL‐1β and the concentration of IL‐1β in iBMDM (Figure S3A,B), which is consistent with the previous study [22]. Due to ASC speck formation being an essential event in NLRP3 inflammasome activation, we lastly determined the formation of the NLRP3 inflammasome by detecting ASC specks and oligomers. Firstly, more ASC dimers and tetramers were detected in the NLRP3_1‐262_ overexpression cell lines stimulated with LPS and ATP under high glucose as well (Figure 4H). Secondly, more ASC specks were observed in the NLRP3_1‐262_ overexpression cell lines stimulated with LPS and ATP under high glucose (Figure 4I,J, Figure S3C).

### 3.5 NLRP3_1_ _‐262_ Truncation Enhances the Activation of NLRP3 Inflammasome by Promoting the Dissociation of Hexokinase‐2 From Mitochondria

**FIGURE 5:**
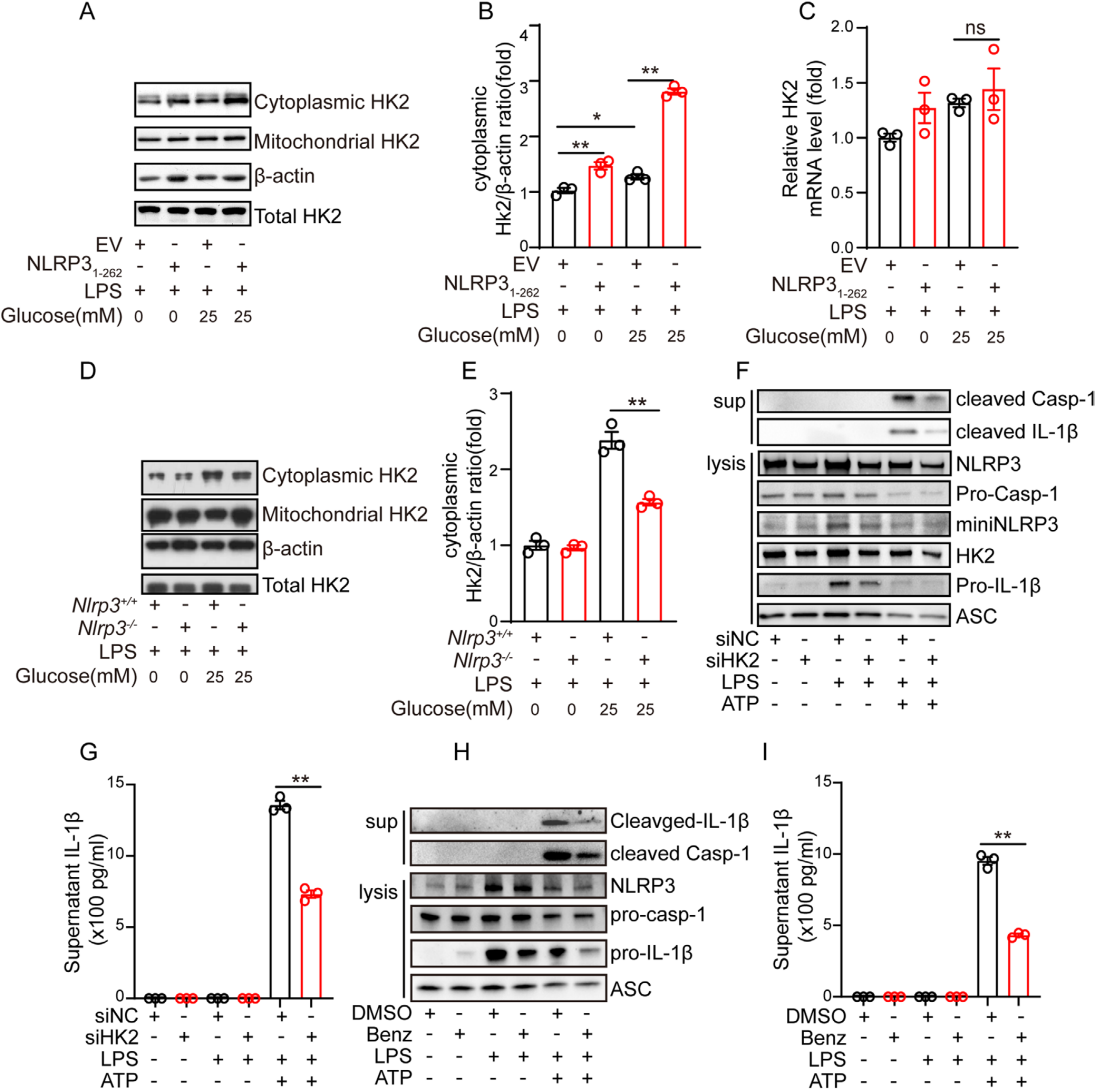
NLRP3_1‐262_ Truncation enhances the activation of NLRP3 inflammasome by promoting the dissociation of Hexokinase‐2 from mitochondria. (A, B) iBMDM cells that stably express empty vector or Flag‐NLRP3_1‐262_ were cultured in medium with 0‐ and 25‐mM glucose for 4 h, then the cells were harvested and the cytoplasmic component and mitochondrial component were separated and the protein levels of HK2 and β‐Actin were detected by western blot. (C) iBMDM cells that stably express empty vector or Flag‐NLRP3_1‐262_ were cultured in medium with 0‐ and 25‐mM glucose for 4 h, then the cells were harvested for detecting the mRNA levels of HK2 by real‐time PCR. (D, E) Wild type and *Nlrp3* KO primary microglia were exposed to LPS (1 μg/mL) in medium with 0‐ and 25‐mM glucose for 4 h, then the cells were harvested and the cytoplasmic component and mitochondrial component were separated and the protein levels of HK2 and β‐Actin were detected by western blot. (F‐G) Primary microglia transfected with negative control siRNA or siRNA against HK2 were exposed to LPS (1 μg/mL) for 4 h at 72 hpi, followed by stimulating with ATP (5 mM) for another 45 min. Then cells were harvested and the protein levels of cleaved IL‐1β, cleaved Casp 1, NLRP3, miniNLRP3, pro‐IL‐1β, HK2, and ASC in cell lysis and medium were detected by western blot (F), the concentration of IL‐1β in the supernatant was determined by ELISA (G). (H, I) Primary microglia pretreated with Benserazide (Benz) or DMSO were exposed to LPS for 4 h, followed by stimulating with ATP for another 45 min. Then cells were harvested and the protein levels of cleaved IL‐1β, cleaved Casp 1, NLRP3, miniNLRP3, pro‐IL‐1β, HK2, and ASC in cell lysis and medium were detected by western blot (H), the concentration of IL‐1β in the supernatant was determined by ELISA (I). (* indicates *p* < 0.05, ** indicates *p* < 0.01 by ANOVA or Student's *t*‐test).

The above results suggest that NLRP3_1‐262_ truncation could enhance the activation of the NLRP3 inflammasome under high glucose conditions; however, its mechanism is unknown. Previous studies reveal that high glucose may affect the activation of NLRP3 by regulating the dissociation of Hexokinase‐2 (HK2) from mitochondria and the expression of P2X purinoceptor 4 (P2RX4) [26, 27, 28]. So, we analyzed the dissociation of HK2 and the expression of P2RX4 in NLRP3_1‐262_ overexpression cell lines, and the results show that high glucose decreased the dissociation of HK2 from mitochondria, while overexpression of NLRP3_1‐262_ increased the dissociation of HK2 from mitochondria (Figure 5A,B). However, no change was observed in the expression of HK2 (Figure 5C) and P2RX4 (Figure S4). While knockout of *Nlrp3* reduced the expression of cytosolic HK2 under high glucose conditions (Figure 5D,E). Then, we further determined whether inhibition of HK2 could affect the activation of the NLRP3 inflammasome under high glucose. The results indicate that the expression of cleaved IL‐1β and cleaved Casp‐1 was significantly reduced in HK2‐silenced cells (Figure 5F). Consistently, the concentration of supernatant IL‐1β was decreased in HK2‐silenced cells (Figure 5G). In addition, inhibition of HK2 with Benserazide (Benz) also decreased the amount of cleaved IL‐1β and cleaved Casp‐1 (Figure 5H), as well as the amount of secreted IL‐1β (Figure 5I).

### 3.6 Conditional Knockout of HK2 in Microglia Could Ameliorate Cerebral I/R Induced Brain Injury and the Activation of NLRP3 Inflammasome Under HGD

**FIGURE 6:**
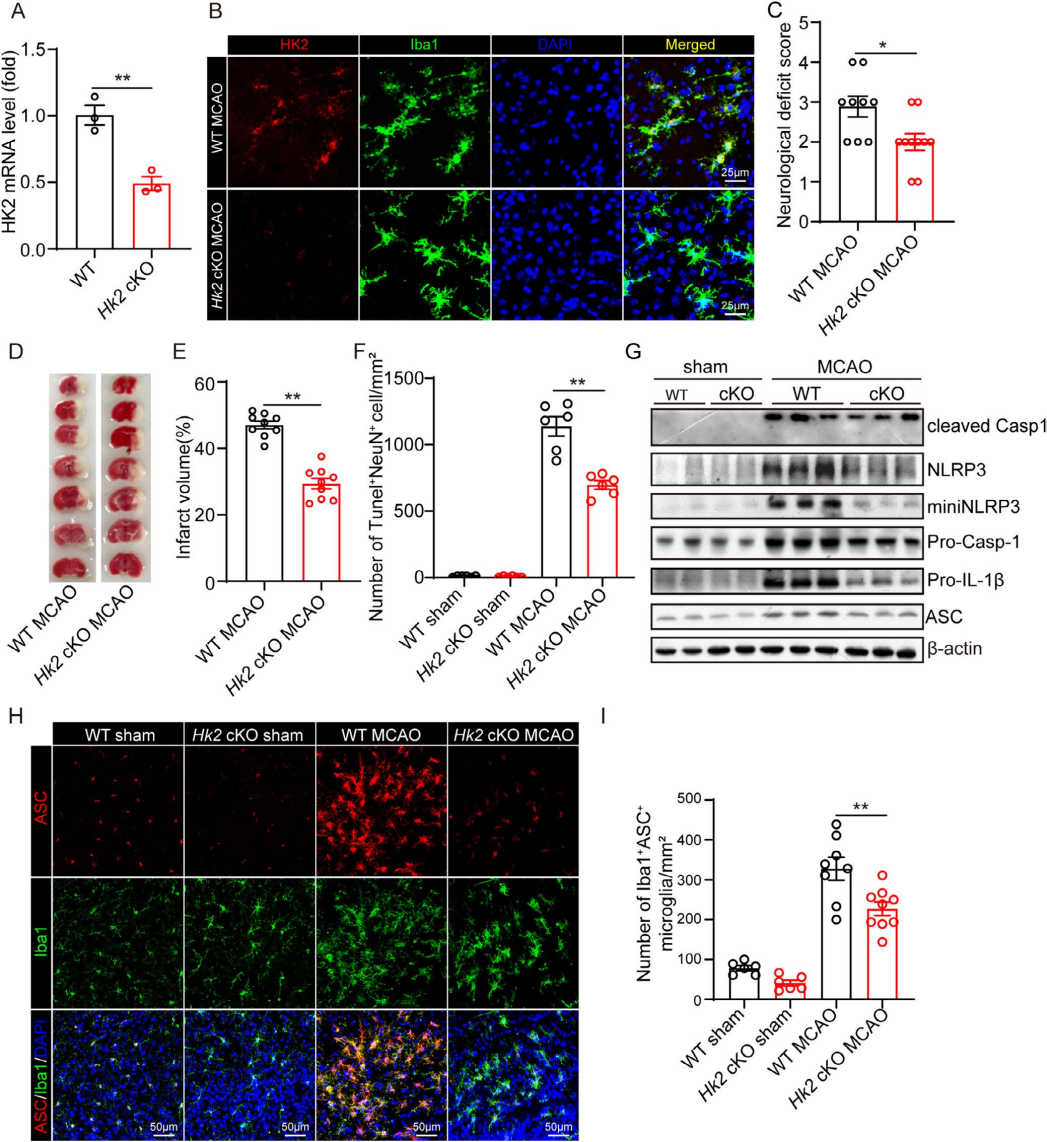
Conditional knockout of HK2 in microglia could ameliorate cerebral I/R induced brain injury and the activation of the NLRP3 inflammasome under HGD. (A) The mRNA levels of Hexokinase 2 (HK2) in primary microglia separated from the brains of wild type and *Hk2* cKO mice 4 weeks post administration of tamoxifen were detected by real‐time PCR. (B) The protein levels of HK2 in microglia in the ischemic penumbra cortex of wild type and *Hk2* cKO mice subjected to tMCAO 24 h post‐reperfusion were detected by indirect immunofluorescent staining and captured by a co‐focal microscope. (C) The neurological deficits of wild type (*n* = 9) and *Hk2* cKO (*n* = 9) mice fed with HGD were assessed 24 h post‐reperfusion. (D‐E) Brain tissue isolated from wild type (*n* = 9) and *Hk2* cKO (*n* = 9) mice fed with HGD 24 h post‐reperfusion was stained with TTC, and infarct volume was determined using ImageJ. (F) The number of TUNEL^+^NeuN^+^ cells in the ischemic penumbra cortex of wild type (*n* = 6) and *Hk2* cKO (*n* = 6) mice fed with HGD 24 h post‐reperfusion was stained with the TUNEL kit and indirect immunofluorescent staining, and the pictures were captured by a co‐focal microscope. (G) The protein levels of NLRP3, miniNLRP3, cleaved Casp 1, pro‐Casp 1, Pro‐IL‐1β, ASC, and β‐Actin in the ischemic penumbra brain tissue from wild type and *Hk2* cKO mice fed with HGD 24 h post‐reperfusion and sham operation were detected by western blot. (H‐I) The number of ASC^+^Iba1^+^ cells in the ischemic penumbra cortex of wild type and *Hk2* cKO mice fed with HGD subjected to tMCAO 24 h post‐reperfusion and sham operation was detected by indirect immunofluorescent staining and captured by a co‐focal microscope. (* indicates *p* < 0.05, ** indicates *p* < 0.01 by ANOVA or Student's *t*‐test).

As HK2 was found to be involved in the miniNLRP3‐mediated activation of the NLRP3 inflammasome, and microglia are the resident immune cells in the brain, we want to ask whether inhibition of microglial HK2 could ameliorate cerebral I/R induced brain injury under HGD. Firstly, we constructed the *Hk2* conditional knockout (*Hk2* cKO) mice by crossing *Hk2* ^*f/f*^ mice with *Cx3cr1* ^*creERT2*^ mice. One month post administration of Tamoxifen, the mice were used for study. The mRNA levels of HK2 were significantly decreased in the microglia purified from *Hk2* cKO mice (Figure 6A), and the number of HK2 and Iba1 double positive cells in the brain from *Hk2* cKO mice was decreased as well (Figure 6B). Then, the wild type (WT) and *Hk2* cKO mice were fed with HGD and the tMCAO was performed 2 days later. The results show that *Hk2* cKO mice displayed a lower neurological deficit score (Figure 6C) and a smaller infarct volume (Figure 6D,E). Consistently, the number of TUNEL positive neurons was decreased in the brain from *Hk2* cKO mice as well (Figure 6F). These results indicate that microglial HK2‐deficit could ameliorate cerebral I/R induced brain injury under HGD. Lastly, we determined whether the activation levels of the NLRP3 inflammasome were affected as well. The results show that the expression of NLRP3, miniNLRP3, pro‐IL‐1β, cleaved IL‐1β, cleaved Casp‐1 and ASC were decreased in the brain from *Hk2* cKO mice that underwent tMCAO and HGD (Figure 6G). In addition, the number of ASC positive microglia was decreased as well (Figure 6H,I).

## 4 Discussion

High blood glucose as one of the risk factors for IS, results in larger infarct volume and more severe sequela post stroke [29]. In addition, high blood glucose is associated with poor functional recovery after I/R [30, 31]. In the current study, we found HGD could increase the blood glucose levels and result in poor outcomes of cerebral I/R‐induced brain injury, including increased infarct volume and apoptotic cell number (Figure 1A–C). Similarly, an acute high‐fat diet could also increase the blood glucose, and worsen cerebral I/R‐induced brain injury [32]. However, its mechanism is obscure. In the current study, our results indicate that the activity of the NLRP3 inflammasome is upregulated in the brain from HGD mice that underwent cerebral I/R. In addition, an N‐terminal truncated NLRP3 (miniNLRP3) band is found in brain tissue that underwent I/R under high glucose conditions, the expression levels of which are positively related to the activation of the NLRP3 inflammasome. Furthermore, the production of miniNLRP3 is dependent on the kinase activity of PKA. The molecular weight of the NLRP3 truncation is close to the N‐terminal 1‐262aa of NLRP3. The activation of NLRP3 is found to be further enhanced under high glucose conditions in cells overexpressing the NLRP3_1‐262_ truncation. Mechanically, overexpression of the NLRP3_1‐262_ truncation promotes the dissociation of HK2 from mitochondria under high glucose conditions, which is essential for the activation of the NLRP3 inflammasome. As a result, knockout of *Nlrp3*, *Pycard* and *Hk2*, could all attenuate cerebral I/R‐induced brain injury under high glucose conditions, which provide therapeutic targets for preventing high blood glucose‐induced poor outcomes in IS patients.

Post‐translational modifications (PTMs), including phosphorylation or ubiquitination of NLRP3, could predispose the protein to proteolytic processing or alter its stability, thereby influencing the abundance of the truncated form [33, 34, 35]. Take for example, a LRR domain‐lacking NLRP3 truncation found in red cells recently, which is proved to mediate the activation of the NLRP3 inflammasome and lytic programmed cell death [36]. Here, we found a 30kD N‐terminal truncation of NLRP3 (miniNLRP3) both in vitro and in vivo. The generation of miniNLRP3 is dependent on the kinase activity of PKA; however, it is not dependent on the S295 of NLRP3, the conserved site that is phosphorylated by PKA. Our further study revealed that PKA‐mediated generation of miniNLRP3 is dependent on the serine protease (Figure S1), despite the specific serine protease having not been uncovered yet. In addition, NLRP3_1‐262_ truncation is very close to the endogenous N‐terminal truncation of NLRP3 produced in the brain from HGD‐fed mice that underwent cerebral I/R and PKA‐mediated cleavage of NLRP3. Overexpression of NLRP3_1‐262_ truncation is found to promote the activation of the NLRP3 inflammasome under high glucose, while inhibition of PKA‐mediated endogenous miniNLRP3 could impair the activation of the NLRP3 inflammasome (Figure 4). These results suggest that the generation of miniNLRP3 is indirectly regulated by PKA, and it is a PTM mediated by the serine protease.

Of note, multiple studies have reported the activation of the NLRP3 inflammasome is enhanced under chronic high glucose exposure [18, 19, 37]. The mechanism includes high glucose upregulating the expression of MARK4 via increasing the expression of ELF3 [18], high glucose‐induced downregulation of cAMP and inhibition of PKA [37]. Here, we found acute high glucose exposure could potentiate the activation of NLRP3 in a PKA kinase‐dependent way, suggesting there is another mechanism for high glucose‐mediated NLRP3 inflammasome activation. High glucose is found to promote the dissociation of HK2 from mitochondria in 2 h, which is a crucial process for the activation of the NLRP3 inflammasome [28, 38]. In the current study, we reveal that NLRP3_1‐262_ truncation enhances the activation of the NLRP3 inflammasome by promoting HK2 dissociation from mitochondria (Figure 5A,B), while knockout of *Nlpr3* decreased high glucose‐induced HK2 dissociation from mitochondria (Figure 5D,E). These results suggest miniNLRP3‐mediated HK2 dissociation from mitochondria is involved in the further activation of the NLRP3 inflammasome under high glucose.

Increasing evidence has shown that glucose metabolism is disturbed in the brain post cerebral I/R and in other brain disease [39, 40, 41]. Several glycolysis‐associated enzymes are upregulated post cerebral I/R, including HK2, pyruvate kinase M2 and L‐lactate dehydrogenase A [39, 42, 43]. Among these enzymes, HK2 is found to be involved in the development of CNS disease. In the development of Alzheimer's disease (AD), HK2 localization to mitochondria is disrupted by Aβ aggregates, which impairs microglial phagocytosis and promotes neuroinflammation [44]. In the development of Parkinson's disease (PD), HK2‐mediated glycolysis exacerbates neuronal damage under rotenone stress, and its inhibition by compounds like gastrodin rescues cell viability [45]. Here, with the help of *Hk2* cKO mice, we demonstrated that inhibition of microglial HK2 could ameliorate high glucose‐induced poor outcomes of ischemic stroke, which is in consistent with a previous study that inhibition of HK2 with lonidamine could attenuate I/R‐induced brain injury in rat [39], suggesting HK2 as a unifying mediator of metabolic and inflammatory dysfunction across neurological diseases.

While our work and others suggest HK2 inhibition as a promising strategy to curb NLRP3 inflammasome activation, potential off‐target effects on neuronal metabolism must be carefully considered. HK2 is expressed not only in immune cells but also in neurons, where it regulates glycolytic flux, mitochondrial stability, and oxidative stress responses [44, 45]. Thus, systemic HK2 inhibition could inadvertently disrupt neuronal energy homeostasis and result in severe side effects. To mitigate the side effects, we could improve the strategies as follows: using nanocarriers or ligands (e.g., TSPO ligands) to deliver HK2 inhibitors preferentially to microglia/macrophages rather than neurons [46]; transient HK2 inhibition during acute inflammatory phases (e.g., post‐stroke) could limit chronic metabolic consequences [47]; coupling HK2 inhibition with antioxidants or metabolic supplements (e.g., lactate) might counteract neuronal energy deficits [48, 49].

In summary, our study illuminates the PKA‐miniNLRP3‐HK2‐NLRP3 inflammasome axis as a key driver of neuroinflammation in hyperglycemic stroke, with broader implications for diabetes and neurodegenerative diseases. While HK2 represents a compelling therapeutic target, its dual roles in immunity and metabolism necessitate precision interventions to avoid off‐target effects. Future studies should directly compare the effects of microglial‐specific versus neuronal‐specific HK2 manipulation in stroke models with comorbid hyperglycemia. Additionally, exploring the upstream proteases generating miniNLRP3 and their relationship to HK2 regulation could unveil more specific targets.

## 5 Conclusions

In summary, our study reveals that high blood glucose exacerbates cerebral I/R‐induced brain injury via promoting the activation of NLRP3. In addition, high glucose induced an N‐terminal truncation of NLRP3, which is close to the N‐terminal 1–262 aa of NLRP3. The generation of miniNLRP3 is mediated by PKA in a serine protease‐dependent manner. Inhibition of PKA‐mediated generation of miniNLRP3 could impair the activation of the NLRP3 inflammasome. Upregulation of NLRP3_1‐262_ truncation could further potentiate the activation of the NLRP3 inflammasome by increasing the dissociation of HK2 from mitochondria. Conventional knockout of *Nlrp3* and *Pycard*, or conditional knockout of *Hk2* in microglia, could all ameliorate cerebral I/R‐induced brain injury under high glucose. Therefore, the PKA‐miniNLRP3‐HK2‐NLRP3 pathway may be a therapeutic target for preventing high glucose‐induced poor outcomes of ischemic stroke.

## Ethics Statement

All animal experiments were approved by the Institutional Animal Care and Use Committee at University of South China and Beijing Institute of Basic Medical Sciences. (Approval Number: 2023–663). This work does not involve any applicable consent to participate.

## Consent

The authors have nothing to report.

## Conflicts of Interest

The authors declare no conflicts of interest.

## Supporting information


**Figure S1:** PKA‐mediated generation of miniNLRP3 is dependent on a serine protease. (A) HEK293T cells transfected with plasmid encoding Flag‐NLRP3 and HA‐PKA were exposed to AEBSF (10, 20, 50, 100 and 200 μM), and the cells were harvested 24 h post transfection, the protein levels of Flag‐NLRP3, Flag‐miniNLRP3, HA‐PKA and GAPDH were determined by western blot. (B) iBMDM pretreated with AEBFS for 30 min were exposed to LPS for 4 h, then the cells were harvested and the protein levels of NLRP3 and GAPDH were determined by western blot. (C) HEK293T cells transfected with plasmid encoding Flag‐NLRP3 and HA‐PKA were exposed to Z‐VAD‐FMK (pan Caspase inhibitor, 10, 20 and 40 μM), VX‐765 (Caspase 1/4 inhibitor, 5, 10 and 20 μM), Ac‐DEVD‐CHO (Caspase 3 inhibitor, 10, 20 and 40 μM), and the cells were harvested 24 h post transfection, the protein levels of Flag‐NLRP3, Flag‐miniNLRP3 and HA‐PKA were determined by western blot.

**Figure S2:** The role of different NLRP3 mutant on PKA‐induced production of miniNLRP3. HEK293T cells were transfected with plasmid encoding HA‐PKA and Flag‐tagged wildtype NLRP3 or NLRP3 mutant that lack amino acid at 233–252, 253–292, 263–282, 293–312 and 313–332, and the protein levels of full‐length proteins and miniNLRP3 were determined by western blot 24 h post transfection.

**Figure S3:** The role of miniNLRP3 on the activation of NLRP3 inflammasome. (A) LPS‐primed iBMDMs were treated with or without H89 (20 μM) 4 h post adding LPS (1 μg/mL), followed by exposing to ATP (5 mM) or Nig (6.7 μM) at 30 min post adding H89 for another 45 min. Then the supernatants and cells were collected and the protein levels of cleaved IL‐1β, cleaved Casp1, pro‐IL‐1β, pro‐Casp1, NLRP3 and ASC were determined by western blot (**A**) and ELISA (B). (C) The iBMDMs that stably expressing NLRP3_1‐262_ or empty vector (EV) were exposed to LPS (1 μg/mL) for 4 h, and followed by treatment with ATP (5 mM) for another 45 min, then the ASC specks were determined by indirect immune staining (the Merge picture for EV + LPS + ATP and NLRP3_1‐262_ + LPS + ATP groups were also displayed in the main Figure 4I).

**Figure S4:** Overexpression of miniNLRP3 has no effect on the expression of P2RX4. iBMDM cells that stably express empty vector or Flag‐NLRP3_1‐262_ were cultured in medium with 0‐ and 25‐mM glucose for 4 h, then the cells were harvested for detecting the mRNA levels of P2RX4 by real‐time PCR.

**Appendix S1:** cns70660‐sup‐0005‐AppendixS1.pdf.

## Data Availability

The data that support the findings of this study are available from the corresponding author upon reasonable request.
