# Supplementary material for: High Glucose Aggravates Cerebral Ischemia/Reperfusion via Truncated NLRP3‐Mediated Hexokinase‐2 Translocation
Source: CNS Neurosci Ther. 2025 Nov 18;31(11):e70660. doi: 10.1111/cns.70660 (PMC12627235; doi:10.1111/cns.70660)
Supplement: Supplementary file 1 — Figure S1: PKA‐mediated generation of miniNLRP3 is dependent on a serine protease. (A) HEK293T cells transfected with plasmid encoding Flag‐NLRP3 and HA‐PKA were exposed to AEBSF (10, 20, 50, 100 and 200 μM), and the cells were harvested 24 h post transfection, the protein levels of Flag‐NLRP3, Flag‐miniNLRP3, HA‐PKA and GAPDH were determined by western blot. (B) iBMDM pretreated with AEBFS for 30 min were exposed to LPS for 4 h, then the cells were harvested and the protein levels of NLRP3 and GAPDH were determined by western blot. (C) HEK293T cells transfected with plasmid encoding Flag‐NLRP3 and HA‐PKA were exposed to Z‐VAD‐FMK (pan Caspase inhibitor, 10, 20 and 40 μM), VX‐765 (Caspase 1/4 inhibitor, 5, 10 and 20 μM), Ac‐DEVD‐CHO (Caspase 3 inhibitor, 10, 20 and 40 μM), and the cells were harvested 24 h post transfection, the protein levels of Flag‐NLRP3, Flag‐miniNLRP3 and HA‐PKA were determined by western blot. [file CNS-31-e70660-s005.zip › FigureS1.docx]

**Figure** **S1.** PKA-mediated generation of miniNLRP3 is dependent on a serine protease. (A) HEK293T cells transfected with plasmid encoding Flag-NLRP3 and HA-PKA were exposed to AEBSF (10, 20, 50, 100 and 200 μM), and the cells were harvested 24 h post transfection, the protein levels of Flag-NLRP3, Flag-miniNLRP3, HA-PKA and GAPDH were determined by western blot. (B) iBMDM pretreated with AEBFS for 30 min were exposed to LPS for 4 h, then the cells were harvested and the protein levels of NLRP3 and GAPDH were determined by western blot. (C) HEK293T cells transfected with plasmid encoding Flag-NLRP3 and HA-PKA were exposed to Z-VAD-FMK (pan Caspase inhibitor, 10, 20 and 40 μM), VX-765 (Caspase 1/4 inhibitor, 5, 10 and 20 μM), Ac-DEVD-CHO (Caspase 3 inhibitor, 10, 20 and 40 μM), and the cells were harvested 24 h post transfection, the protein levels of Flag-NLRP3, Flag-miniNLRP3 and HA-PKA were determined by western blot.
