# Supplementary figures and images for: High Glucose Aggravates Cerebral Ischemia/Reperfusion via Truncated NLRP3‐Mediated Hexokinase‐2 Translocation
Source: CNS Neurosci Ther. 2025 Nov 18;31(11):e70660. doi: 10.1111/cns.70660 (PMC12627235; doi:10.1111/cns.70660)

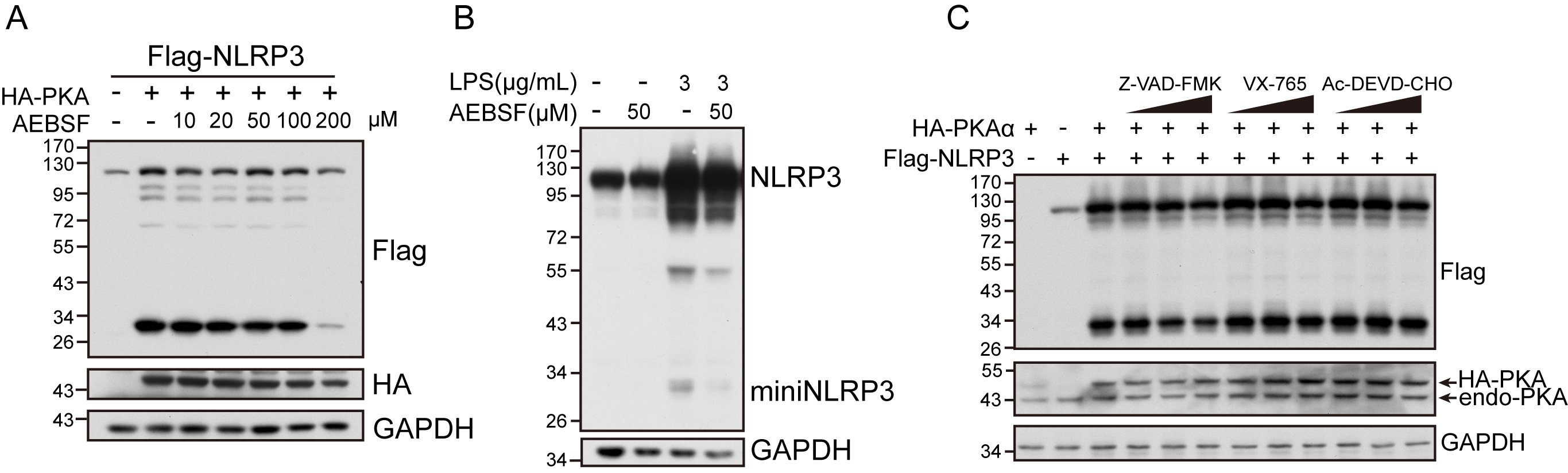

Supplement: Supplementary file 1 — Figure S1: PKA‐mediated generation of miniNLRP3 is dependent on a serine protease. (A) HEK293T cells transfected with plasmid encoding Flag‐NLRP3 and HA‐PKA were exposed to AEBSF (10, 20, 50, 100 and 200 μM), and the cells were harvested 24 h post transfection, the protein levels of Flag‐NLRP3, Flag‐miniNLRP3, HA‐PKA and GAPDH were determined by western blot. (B) iBMDM pretreated with AEBFS for 30 min were exposed to LPS for 4 h, then the cells were harvested and the protein levels of NLRP3 and GAPDH were determined by western blot. (C) HEK293T cells transfected with plasmid encoding Flag‐NLRP3 and HA‐PKA were exposed to Z‐VAD‐FMK (pan Caspase inhibitor, 10, 20 and 40 μM), VX‐765 (Caspase 1/4 inhibitor, 5, 10 and 20 μM), Ac‐DEVD‐CHO (Caspase 3 inhibitor, 10, 20 and 40 μM), and the cells were harvested 24 h post transfection, the protein levels of Flag‐NLRP3, Flag‐miniNLRP3 and HA‐PKA were determined by western blot. [file CNS-31-e70660-s005.zip › cns70660-sup-0001-FigureS1@Fig S1.tif]

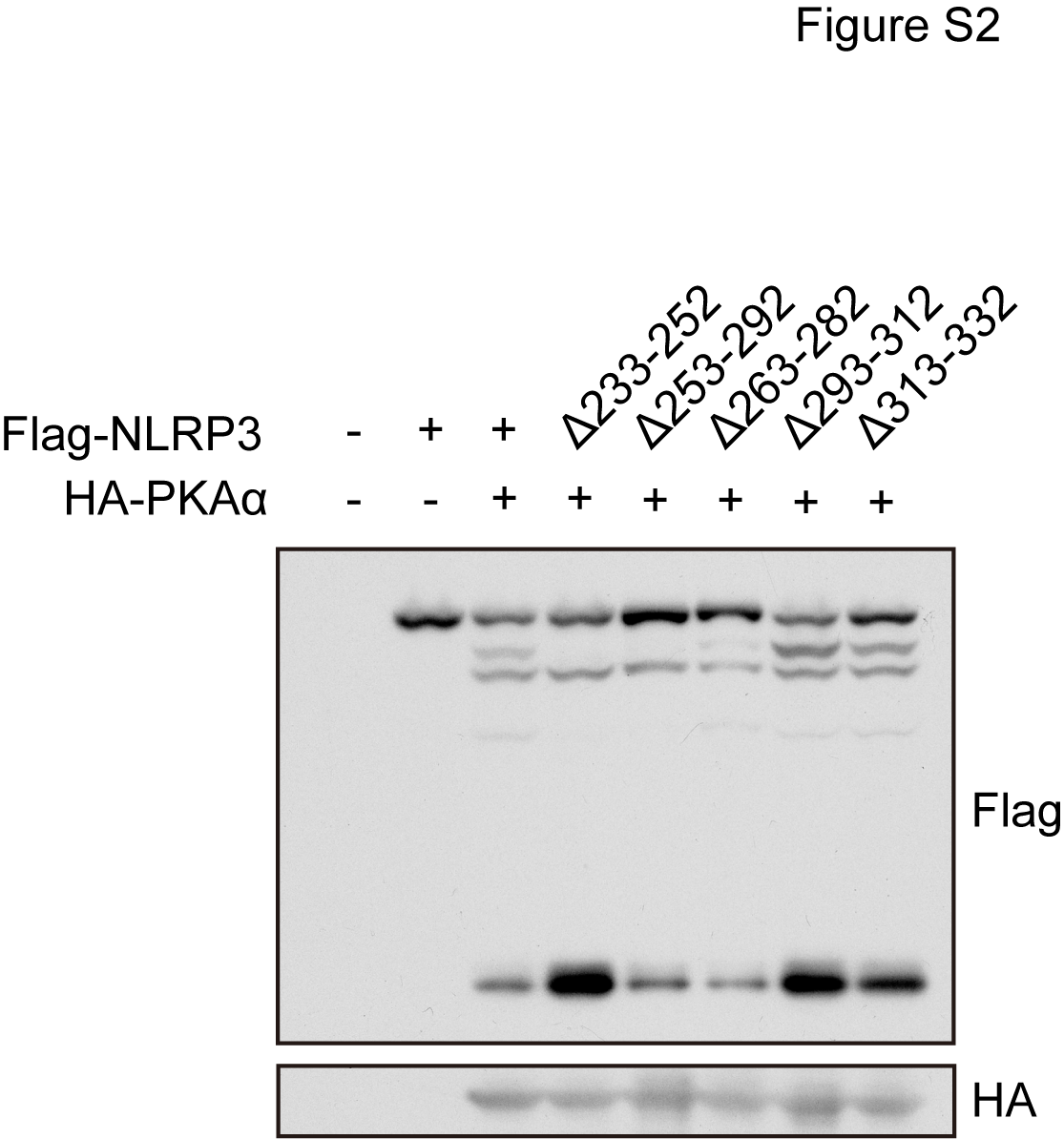

Supplement: Supplementary file 2 — Figure S2: The role of different NLRP3 mutant on PKA‐induced production of miniNLRP3. HEK293T cells were transfected with plasmid encoding HA‐PKA and Flag‐tagged wildtype NLRP3 or NLRP3 mutant that lack amino acid at 233–252, 253–292, 263–282, 293–312 and 313–332, and the protein levels of full‐length proteins and miniNLRP3 were determined by western blot 24 h post transfection. [file CNS-31-e70660-s001.zip › cns70660-sup-0003-FigureS2@Fig S2.tif]

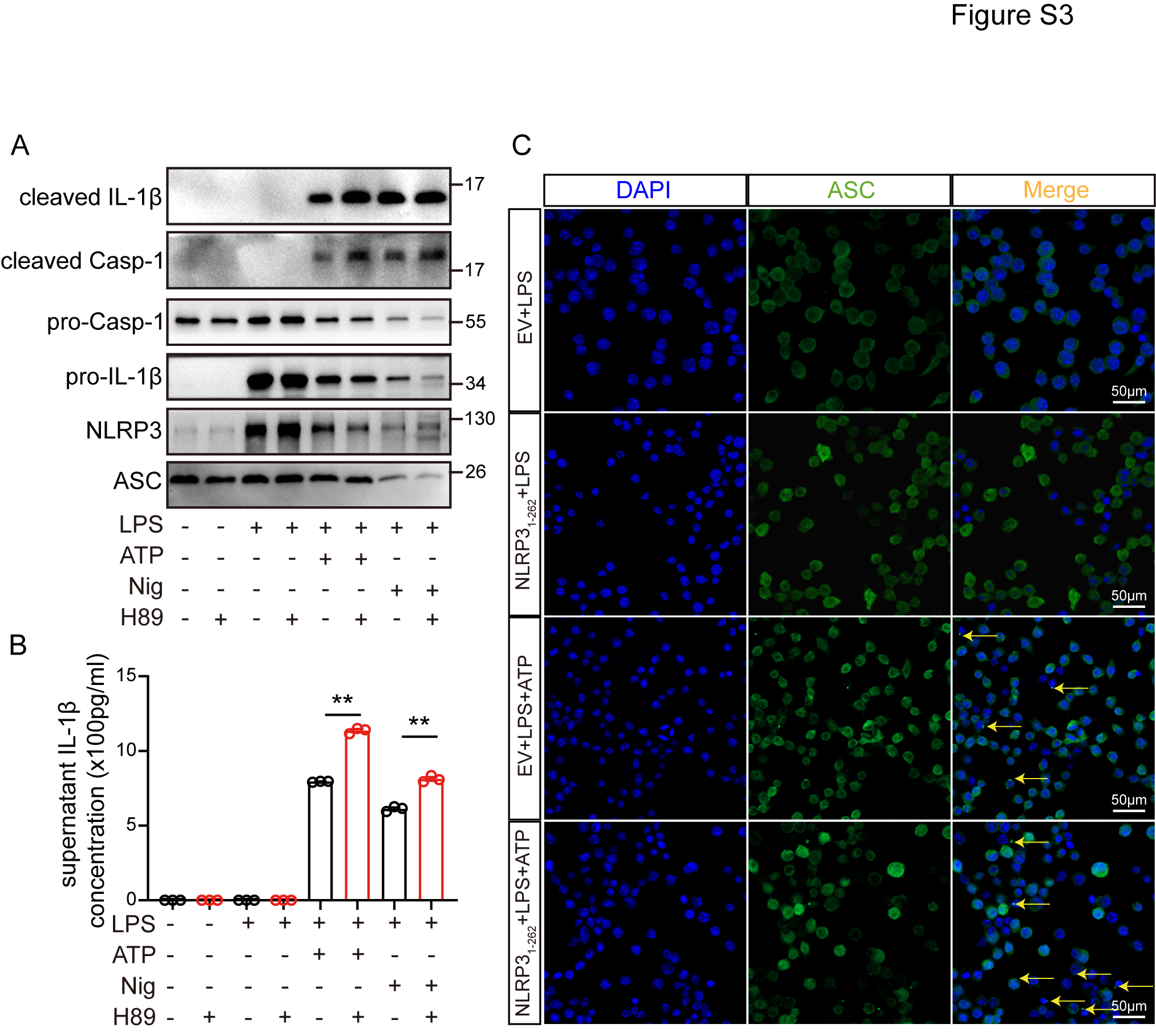

Supplement: Supplementary file 3 — Figure S3: The role of miniNLRP3 on the activation of NLRP3 inflammasome. (A) LPS‐primed iBMDMs were treated with or without H89 (20 μM) 4 h post adding LPS (1 μg/mL), followed by exposing to ATP (5 mM) or Nig (6.7 μM) at 30 min post adding H89 for another 45 min. Then the supernatants and cells were collected and the protein levels of cleaved IL‐1β, cleaved Casp1, pro‐IL‐1β, pro‐Casp1, NLRP3 and ASC were determined by western blot (A) and ELISA (B). (C) The iBMDMs that stably expressing NLRP31‐262 or empty vector (EV) were exposed to LPS (1 μg/mL) for 4 h, and followed by treatment with ATP (5 mM) for another 45 min, then the ASC specks were determined by indirect immune staining (the Merge picture for EV + LPS + ATP and NLRP31‐262 + LPS + ATP groups were also displayed in the main Figure 4I). [file CNS-31-e70660-s002.zip › cns70660-sup-0004-FigureS3@Fig S3.tif]

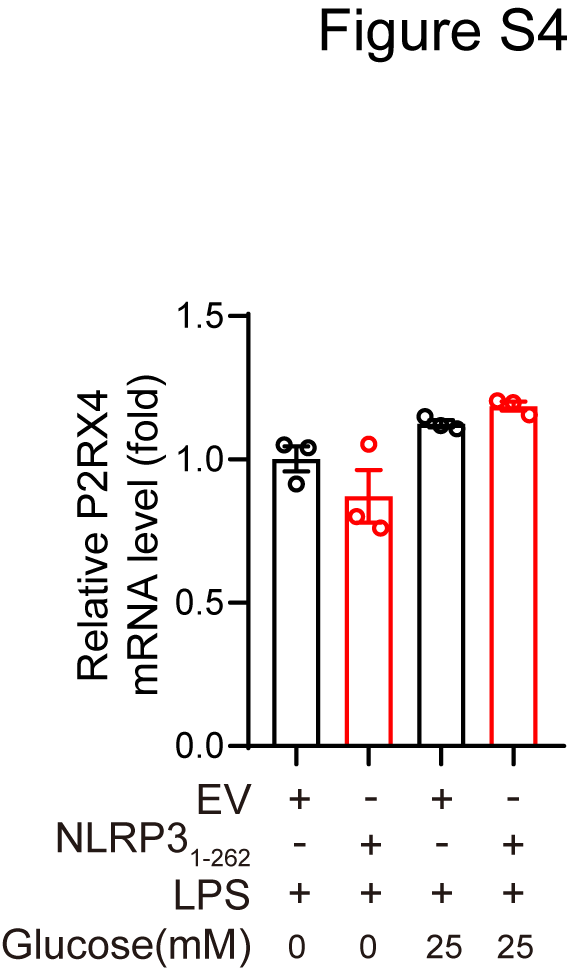

Supplement: Supplementary file 4 — Figure S4: Overexpression of miniNLRP3 has no effect on the expression of P2RX4. iBMDM cells that stably express empty vector or Flag‐NLRP31‐262 were cultured in medium with 0‐ and 25‐mM glucose for 4 h, then the cells were harvested for detecting the mRNA levels of P2RX4 by real‐time PCR. [file CNS-31-e70660-s004.zip › cns70660-sup-0005-FigureS4@Fig S4.tif]
