# Supplementary material for: High Glucose Aggravates Cerebral Ischemia/Reperfusion via Truncated NLRP3‐Mediated Hexokinase‐2 Translocation
Source: CNS Neurosci Ther. 2025 Nov 18;31(11):e70660. doi: 10.1111/cns.70660 (PMC12627235; doi:10.1111/cns.70660)
Supplement: Supplementary file 2 — Figure S2: The role of different NLRP3 mutant on PKA‐induced production of miniNLRP3. HEK293T cells were transfected with plasmid encoding HA‐PKA and Flag‐tagged wildtype NLRP3 or NLRP3 mutant that lack amino acid at 233–252, 253–292, 263–282, 293–312 and 313–332, and the protein levels of full‐length proteins and miniNLRP3 were determined by western blot 24 h post transfection. [file CNS-31-e70660-s001.zip › Figure S2.docx]

**Figure** **S2.** The role of different NLRP3 mutant on PKA-induced production of miniNLRP3. HEK293T cells were transfected with plasmid encoding HA-PKA and Flag-tagged wildtype NLRP3 or NLRP3 mutant that lack amino acid at 233–252, 253–292, 263–282, 293–312 and 313–332, and the protein levels of full-length proteins and miniNLRP3 were determined by western blot 24 h post transfection.
