# Supplementary material for: High Glucose Aggravates Cerebral Ischemia/Reperfusion via Truncated NLRP3‐Mediated Hexokinase‐2 Translocation
Source: CNS Neurosci Ther. 2025 Nov 18;31(11):e70660. doi: 10.1111/cns.70660 (PMC12627235; doi:10.1111/cns.70660)
Supplement: Supplementary file 3 — Figure S3: The role of miniNLRP3 on the activation of NLRP3 inflammasome. (A) LPS‐primed iBMDMs were treated with or without H89 (20 μM) 4 h post adding LPS (1 μg/mL), followed by exposing to ATP (5 mM) or Nig (6.7 μM) at 30 min post adding H89 for another 45 min. Then the supernatants and cells were collected and the protein levels of cleaved IL‐1β, cleaved Casp1, pro‐IL‐1β, pro‐Casp1, NLRP3 and ASC were determined by western blot (A) and ELISA (B). (C) The iBMDMs that stably expressing NLRP31‐262 or empty vector (EV) were exposed to LPS (1 μg/mL) for 4 h, and followed by treatment with ATP (5 mM) for another 45 min, then the ASC specks were determined by indirect immune staining (the Merge picture for EV + LPS + ATP and NLRP31‐262 + LPS + ATP groups were also displayed in the main Figure 4I). [file CNS-31-e70660-s002.zip › Figure S3.docx]

**Figure** **S3.** The role of miniNLRP3 on the activation of NLRP3 inflammasome. (A) LPS-primed iBMDMs were treated with or without H89 (20 μM) 4 h post adding LPS (1 μg/mL), followed by exposing to ATP (5 mM) or Nig (6.7 μM) at 30 min post adding H89 for another 45 min. Then the supernatants and cells were collected and the protein levels of cleaved IL-1β, cleaved Casp1, pro-IL-1β, pro-Casp1, NLRP3 and ASC were determined by western blot (**A**) and ELISA (B). (C) The iBMDMs that stably expressing NLRP3_1-262_ or empty vector (EV) were exposed to LPS (1 μg/mL) for 4 h, and followed by treatment with ATP (5 mM) for another 45 min, then the ASC specks were determined by indirect immune staining (the Merge picture for EV + LPS + ATP and NLRP3_1-262_ + LPS + ATP groups were also displayed in the main Figure 4I).
