# Supplementary material for: High Glucose Aggravates Cerebral Ischemia/Reperfusion via Truncated NLRP3‐Mediated Hexokinase‐2 Translocation
Source: CNS Neurosci Ther. 2025 Nov 18;31(11):e70660. doi: 10.1111/cns.70660 (PMC12627235; doi:10.1111/cns.70660)
Supplement: Supplementary file 4 — Figure S4: Overexpression of miniNLRP3 has no effect on the expression of P2RX4. iBMDM cells that stably express empty vector or Flag‐NLRP31‐262 were cultured in medium with 0‐ and 25‐mM glucose for 4 h, then the cells were harvested for detecting the mRNA levels of P2RX4 by real‐time PCR. [file CNS-31-e70660-s004.zip › Figure S4.docx]

**Figure** **S4.** Overexpression of miniNLRP3 has no effect on the expression of P2RX4. iBMDM cells that stably express empty vector or Flag-NLRP3_1-262_ were cultured in medium with 0- and 25-mM glucose for 4 h, then the cells were harvested for detecting the mRNA levels of P2RX4 by real-time PCR.
