# Supplementary material for: High Glucose Aggravates Cerebral Ischemia/Reperfusion via Truncated NLRP3‐Mediated Hexokinase‐2 Translocation
Source: CNS Neurosci Ther. 2025 Nov 18;31(11):e70660. doi: 10.1111/cns.70660 (PMC12627235; doi:10.1111/cns.70660)

Fig1

Full unedited gel/blot for Figure 1F

actin

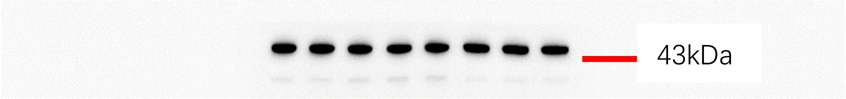

Pro-casp-1

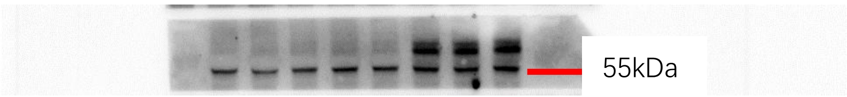

GSDMD

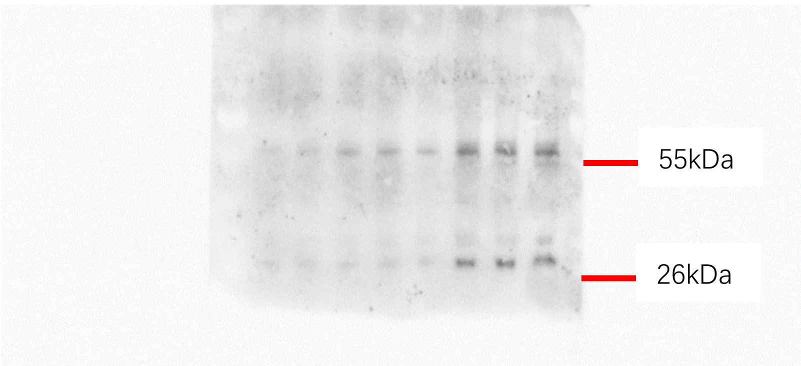

NLRP3

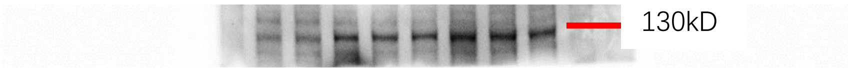

PRO-IL-1β;ASC

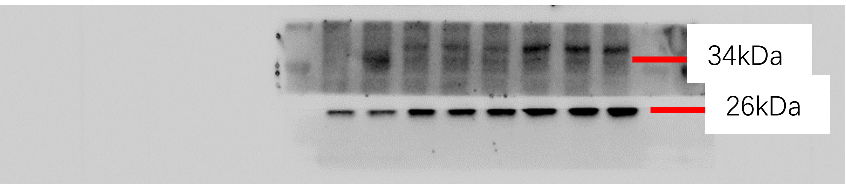

Fig2

Full unedited gel/blot for Figure 2F

ASC

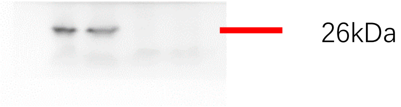

Actin

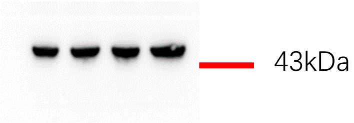

Fig2A

Nlrp3

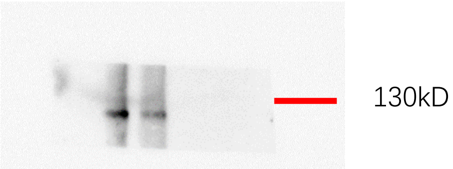

Actin

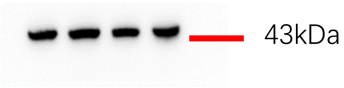

Fig3

Full unedited gel/blot for Figure 3A

Nlrp3

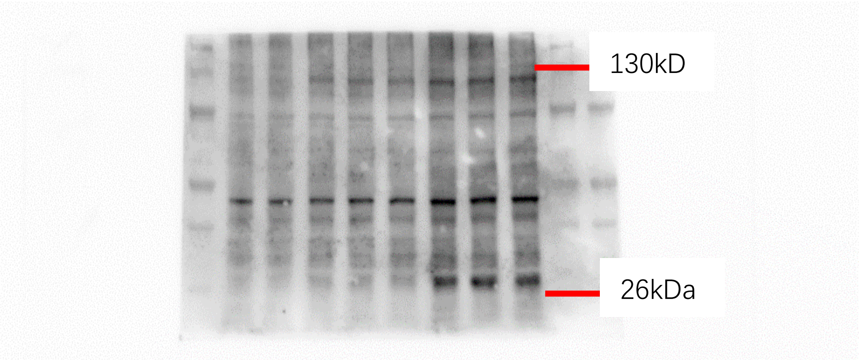

Actin

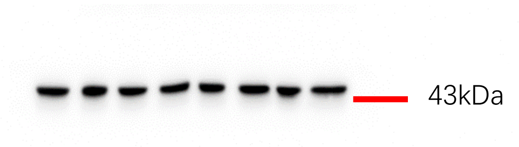

Full unedited gel/blot for Figure 3B

Tubulin

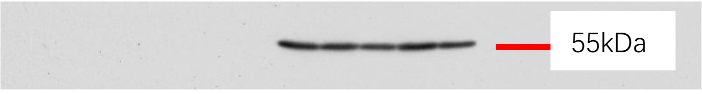

Nlrp3(SE)

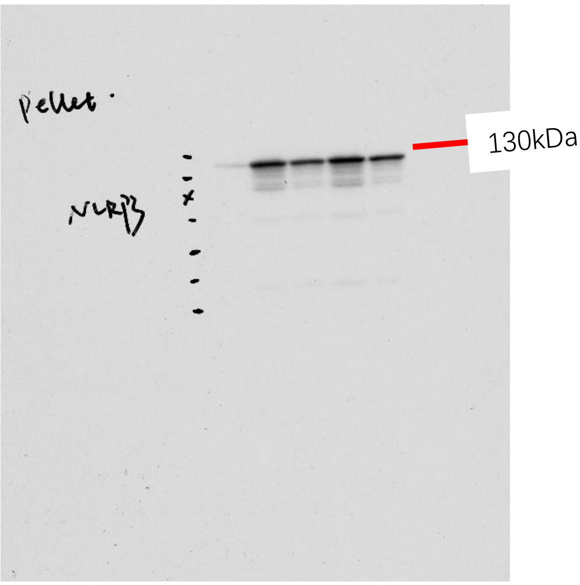

Nlrp3

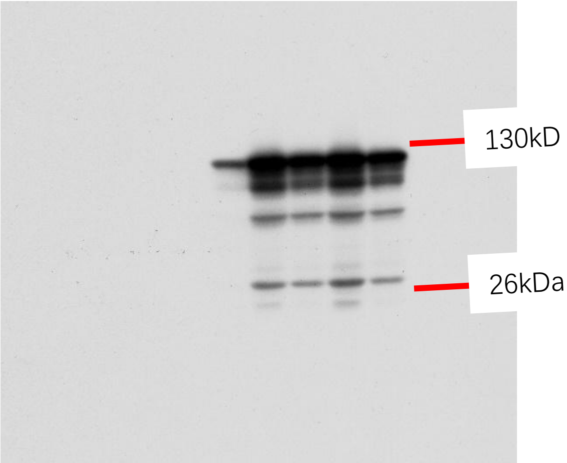

Pro-IL-1β

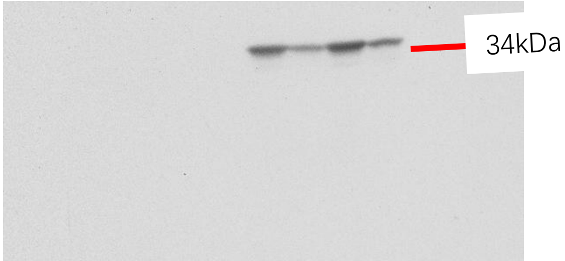

cleaved-IL-1β

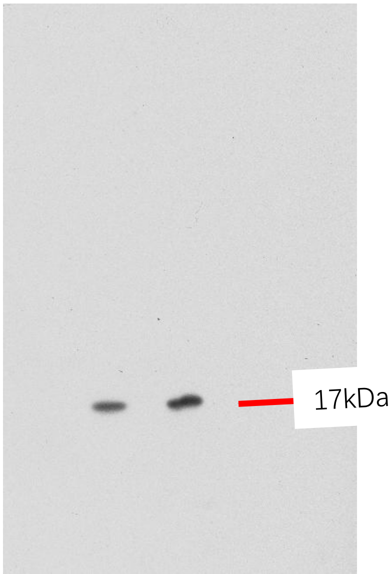
17kDa

Full unedited gel/blot for Figure 3C

NLRP3

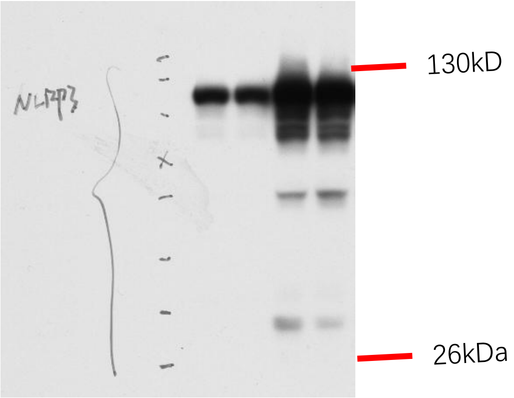

GAPDH

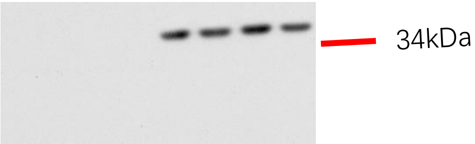

Full unedited gel/blot for Figure 3D

HA

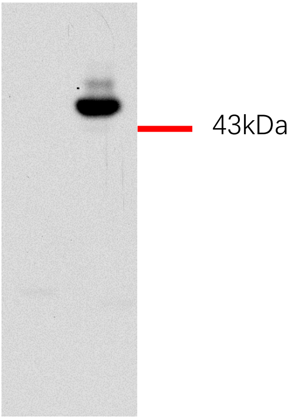

Flag

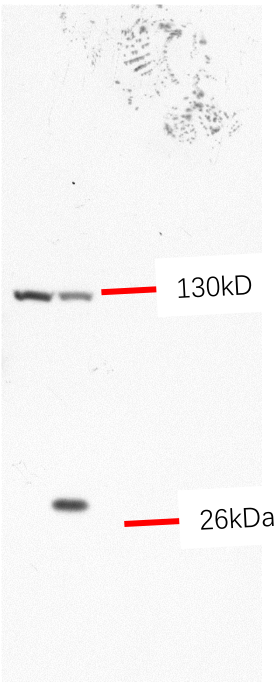

pFlag-NLRP3

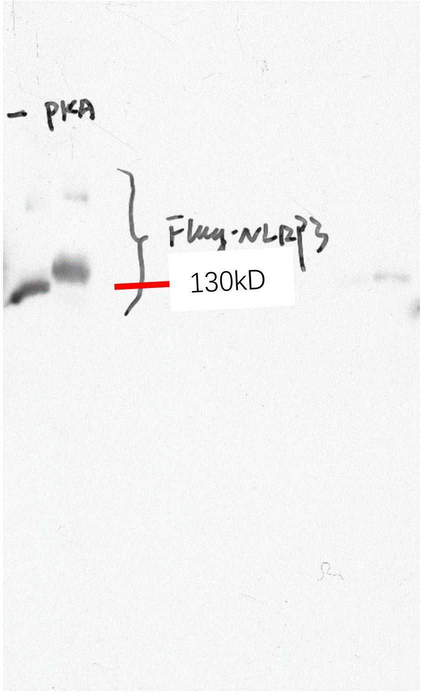

PKA
Flag-NLRP3
130kD

Full unedited gel/blot for Figure 3E

Flag

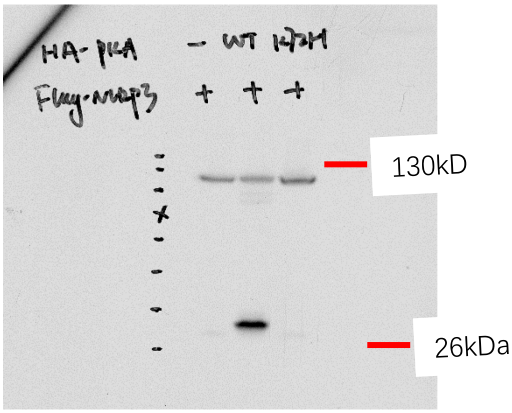

HA

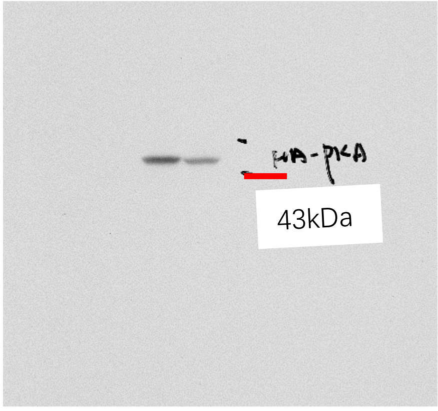

Full unedited gel/blot for Figure 3F

Flag

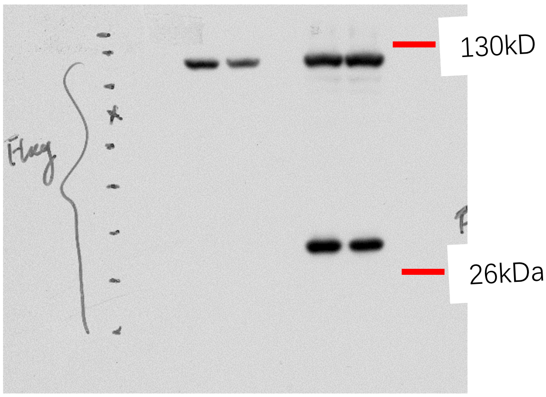

Full unedited gel/blot for Figure 3G

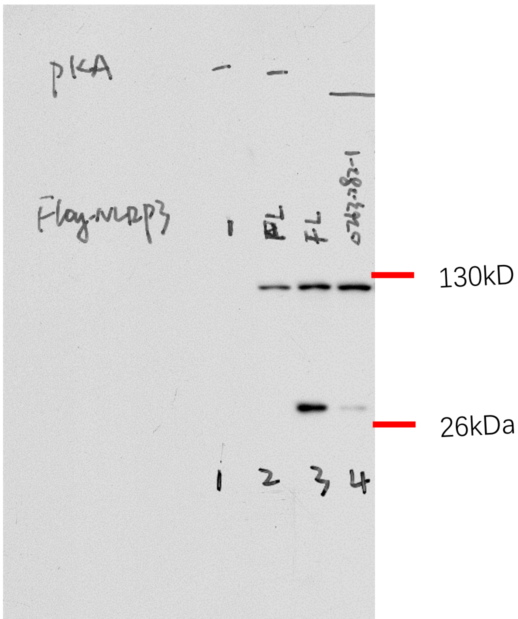

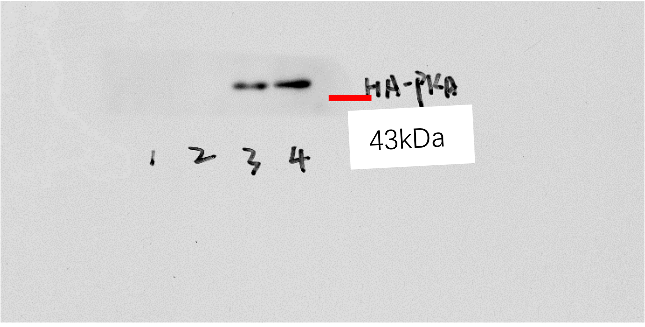

Full unedited gel/blot for Figure 3H

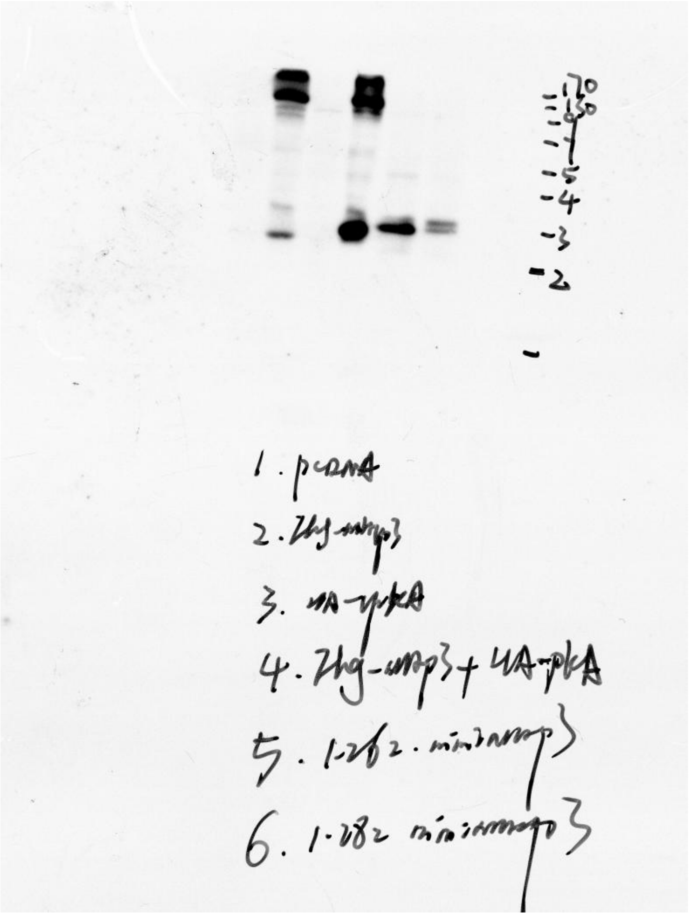

Fig4

Full unedited gel/blot for Figure 4A

NLRP3

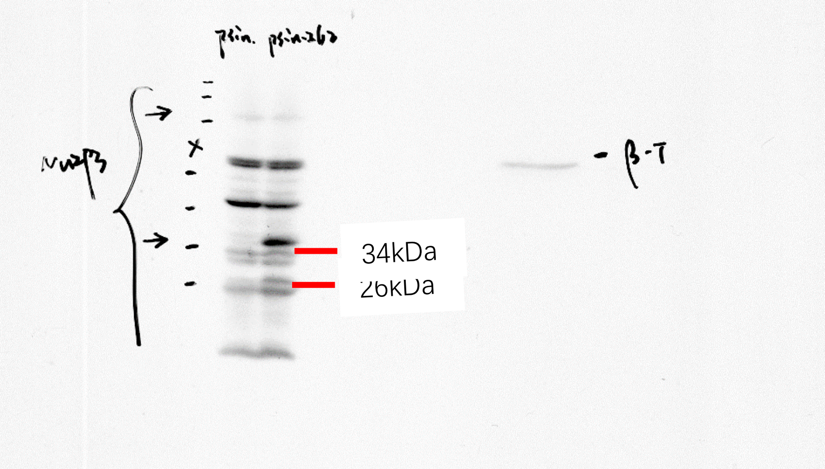

Tubulin

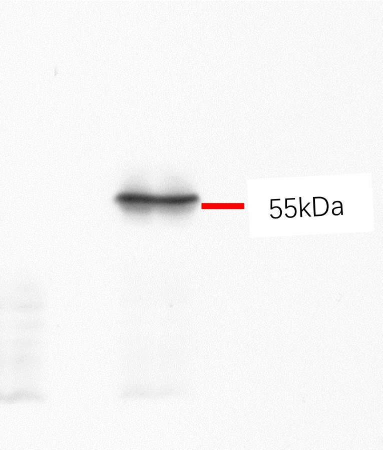

Full unedited gel/blot for Figure 4B

Cleaved- casp-1

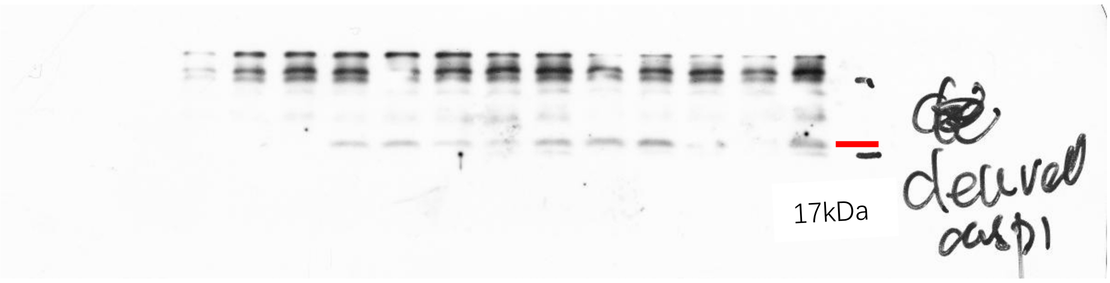

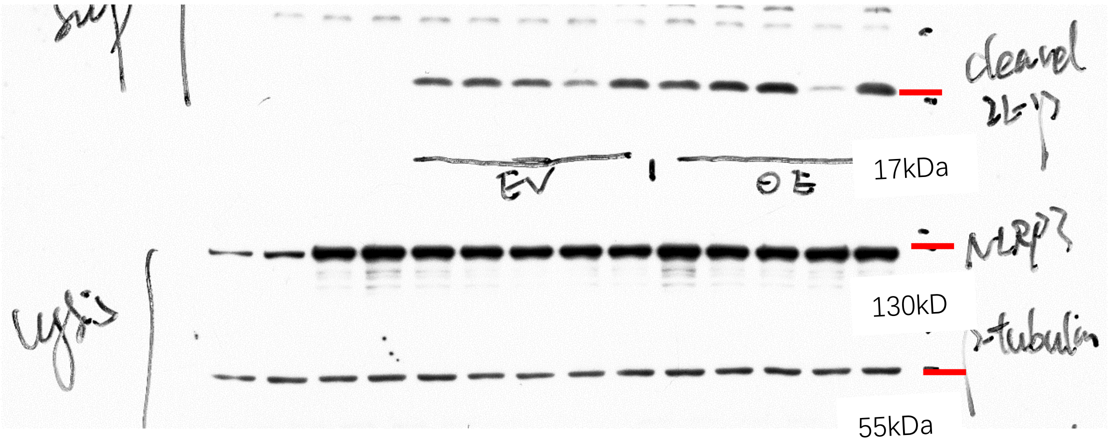

Nlrp3(SE); pro-IL-1β

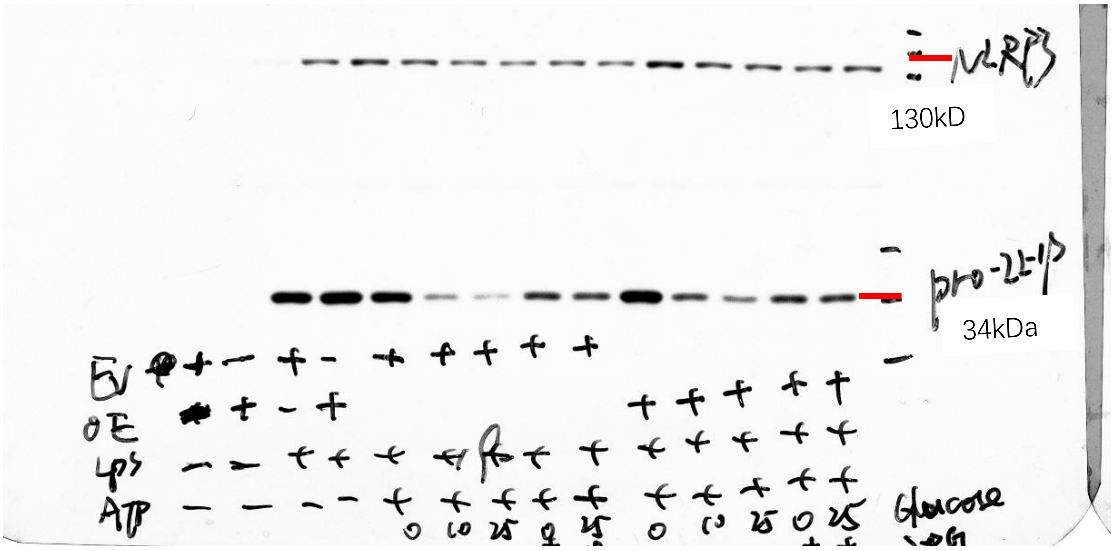

Full unedited gel/blot for Figure 4E

NLRP3

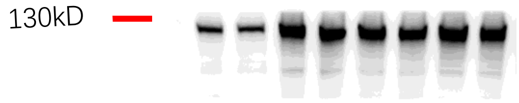

Pro-caspase-1

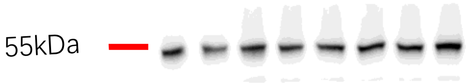

cleaved-IL-1β

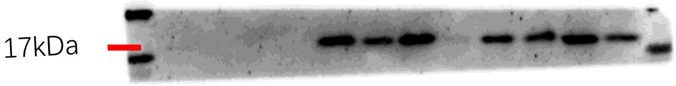

IL-1β

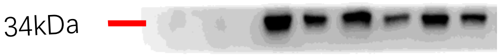

ASC

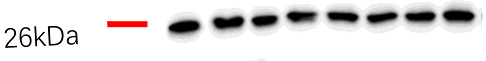

Actin

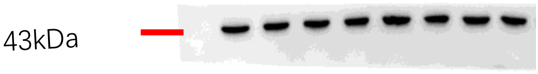

MiniNLRP3

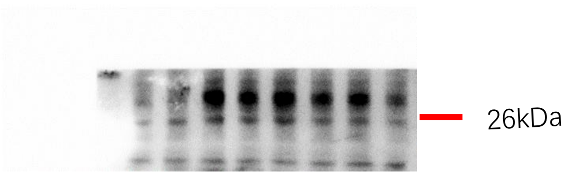

Full unedited gel/blot for Figure 4H

ASC

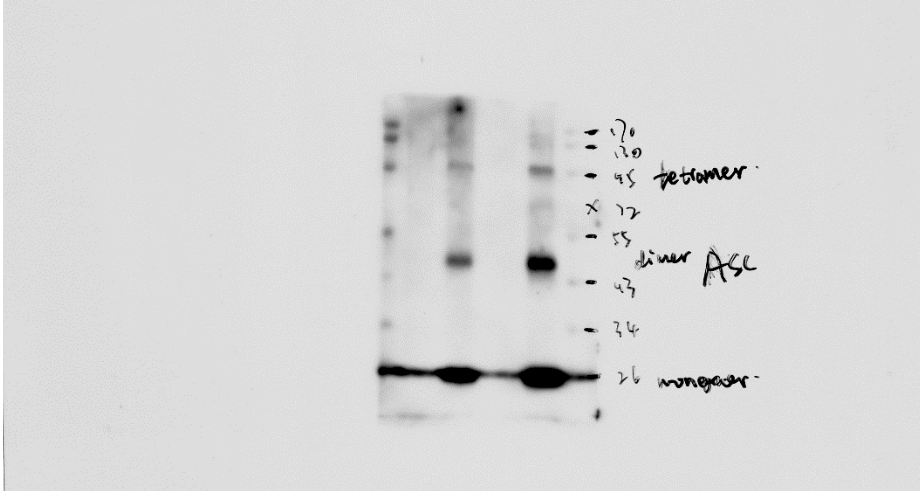

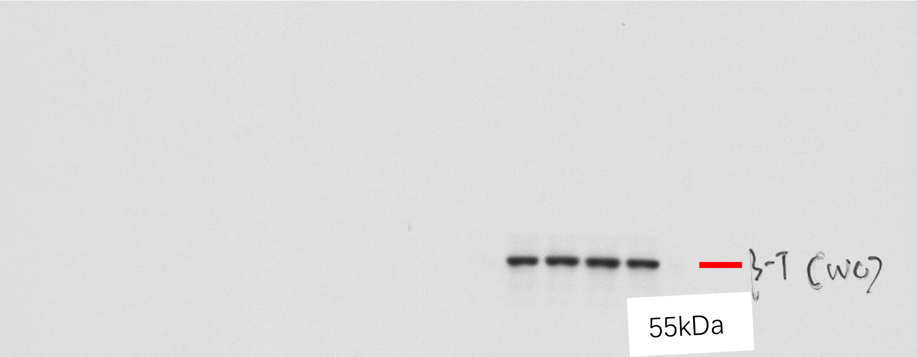

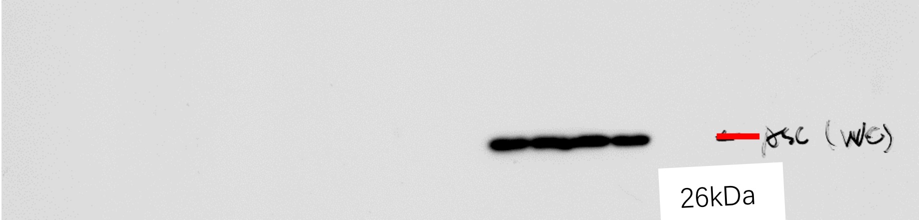

Fig5

Full unedited gel/blot for Figure 5A

Cytoplasmichk2

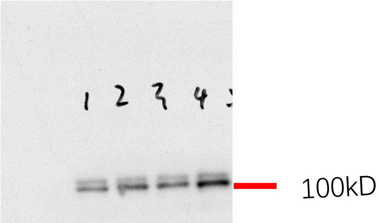

Total HK2

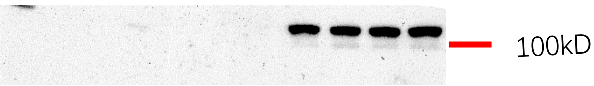

mitochondrial hk2

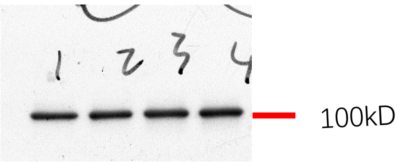

Actin

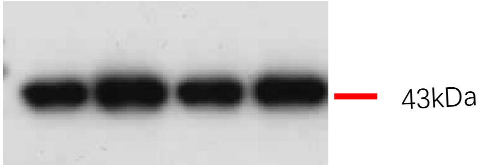

Full unedited gel/blot for Figure 5D

Cytoplasmichk2

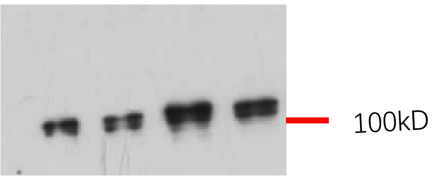

mitochondrial hk2

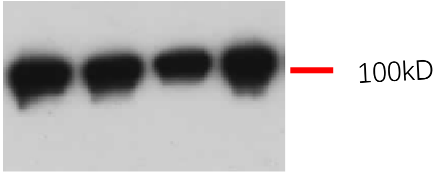

β-actin

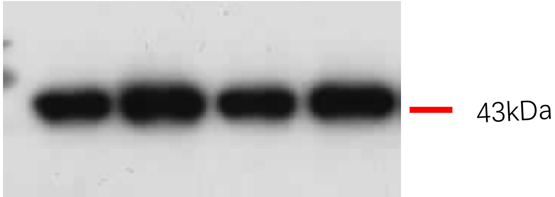

TotalHK2

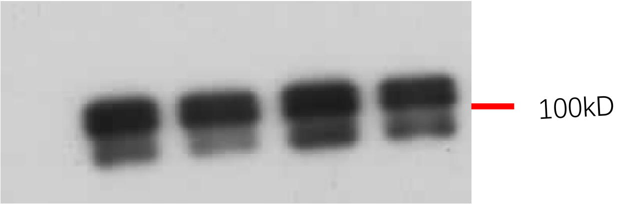

Full unedited gel/blot for Figure 5F

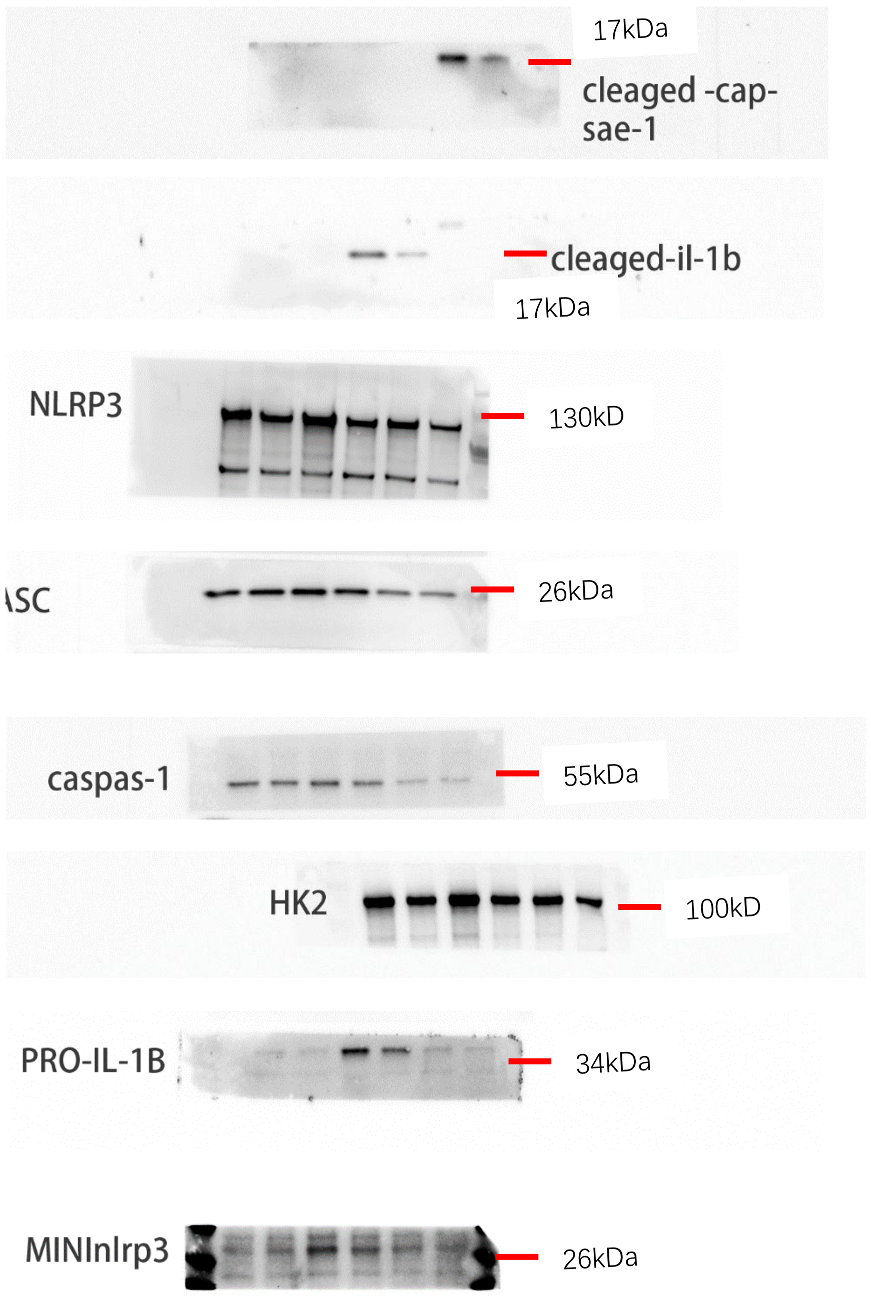

Full unedited gel/blot for Figure 5H

NLRP3

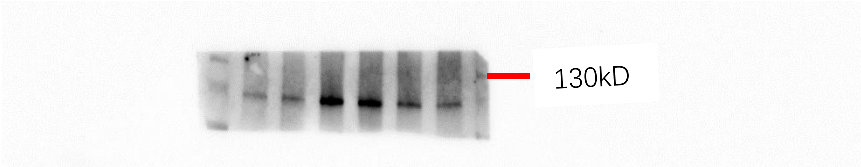

Pro-CAPS-1; Pro-IL-1β; ASC

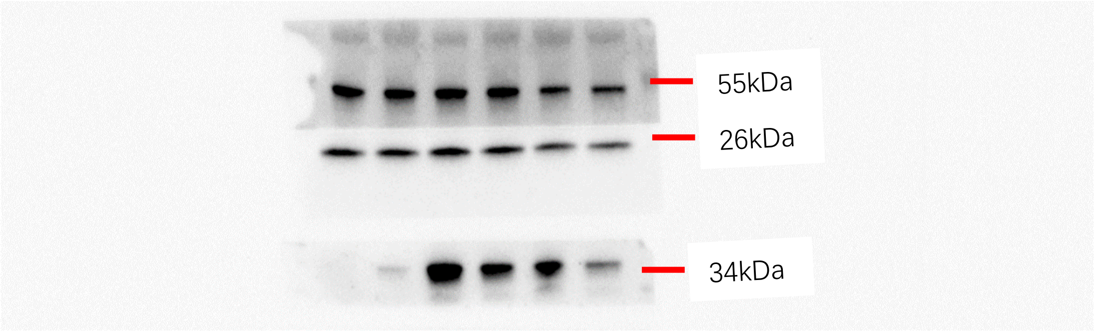

cleaved IL-1β

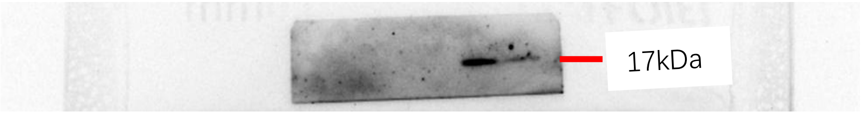

cleaved Casp 1

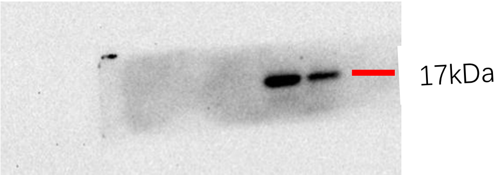

Fig6

Full unedited gel/blot for Figure 6G

β-actin

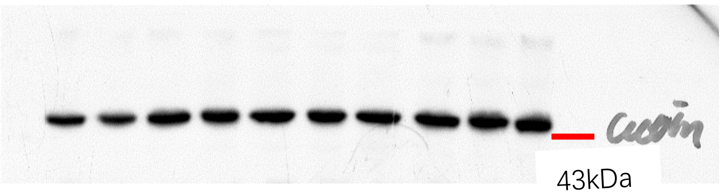

ASC

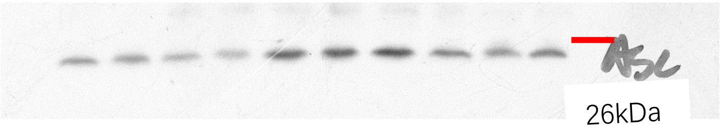

pro-Casp 1

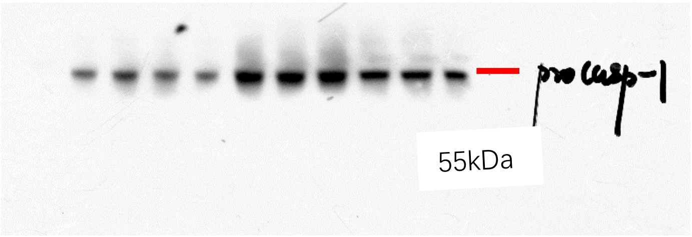

Cleavage-Casp 1

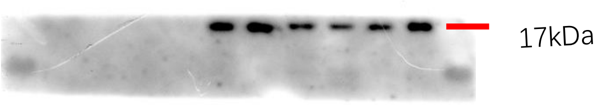

Pro-IL-1b

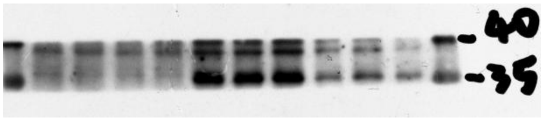

MiniNLRP3

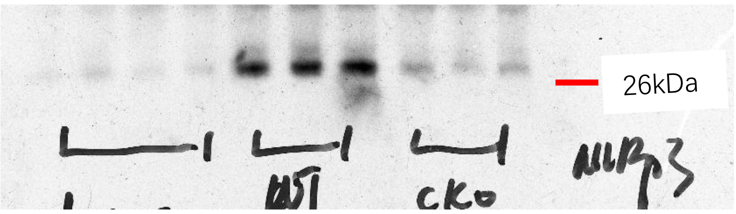

NLRP3

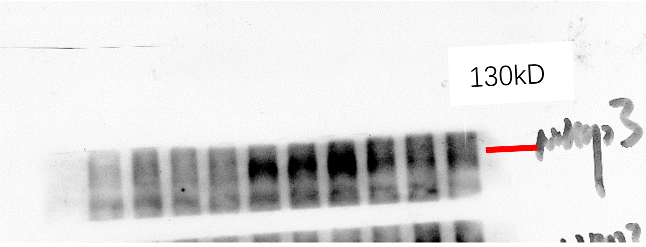
130kD

FigS1

Full unedited gel/blot for Figure S1A

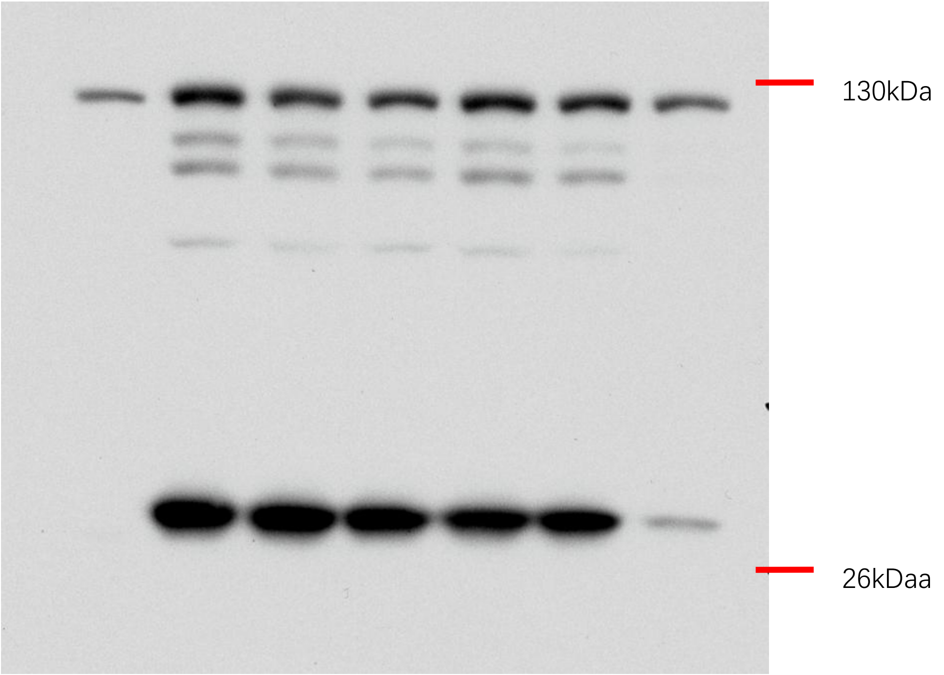

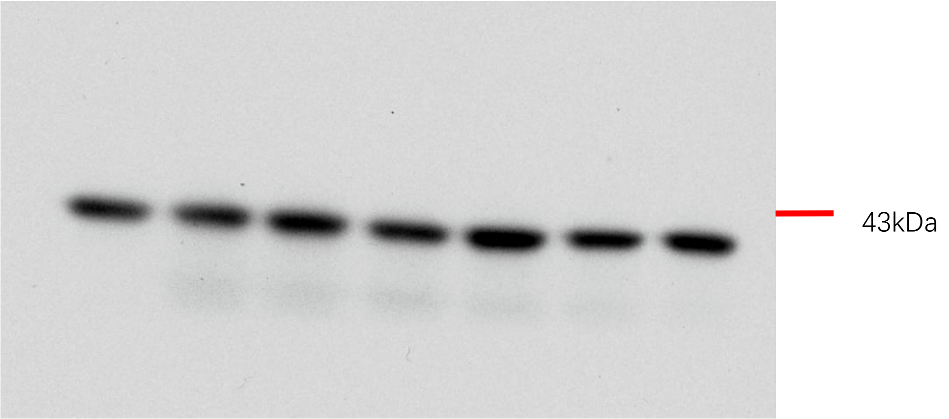

Full unedited gel/blot for Figure S1B

NLRP3

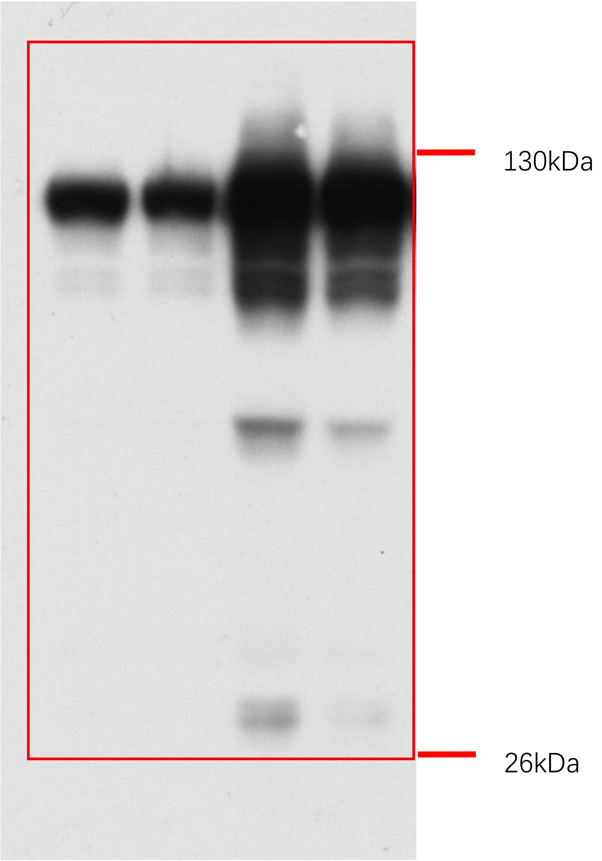

GAPDH

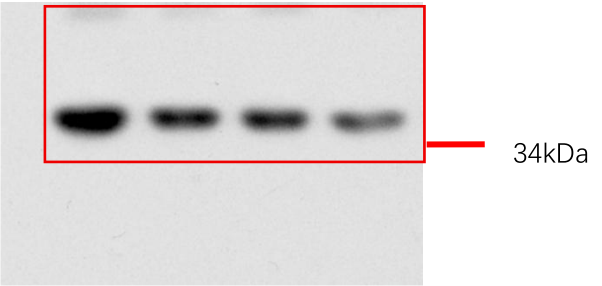

Full unedited gel/blot for Figure S1C

Flag

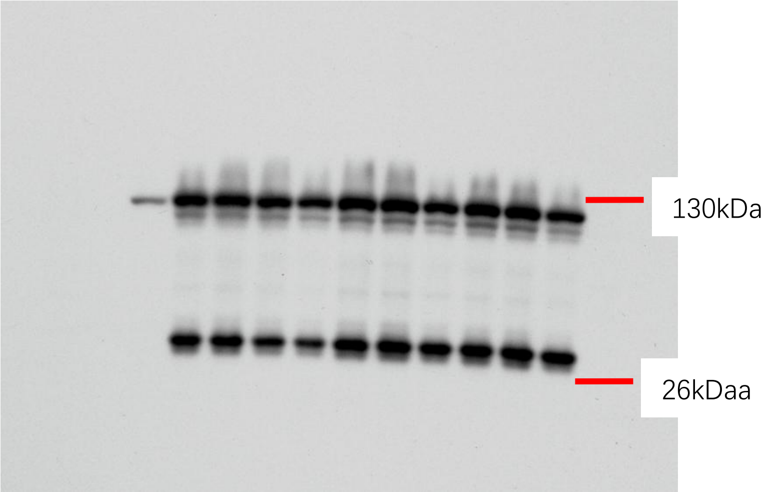

PKA(HA-PKA and endogenous PKA)

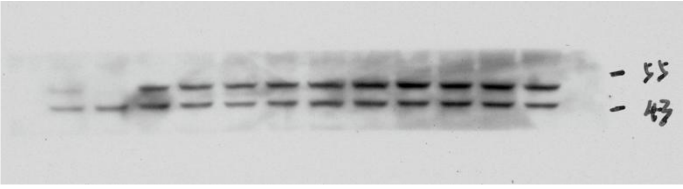

GAPDH

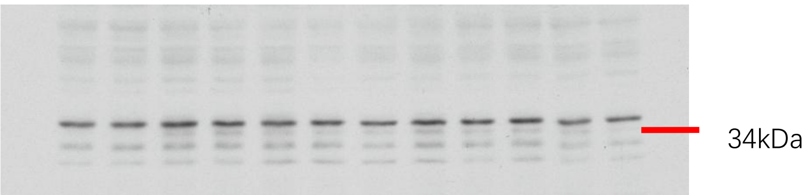

FigS2
Full unedited gel/blot for Figure S2
Flag

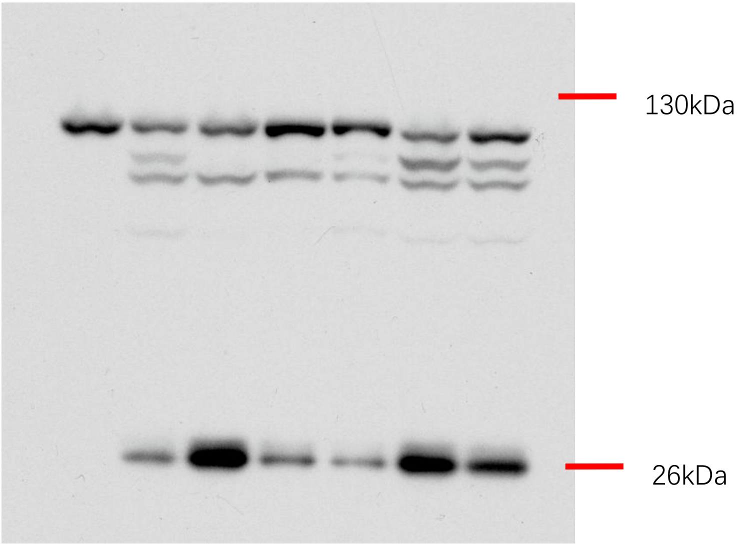

HA

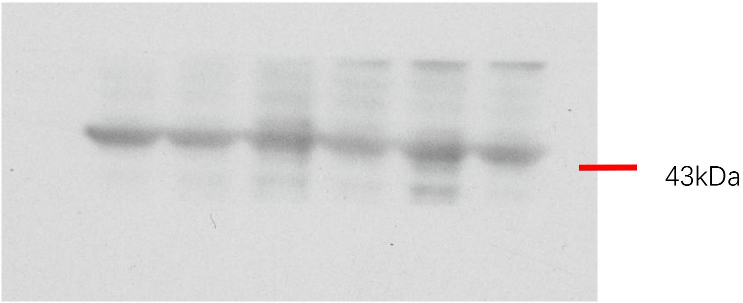

FigS3

Full unedited gel/blot for Figure S3A

Cleaved IL-1β

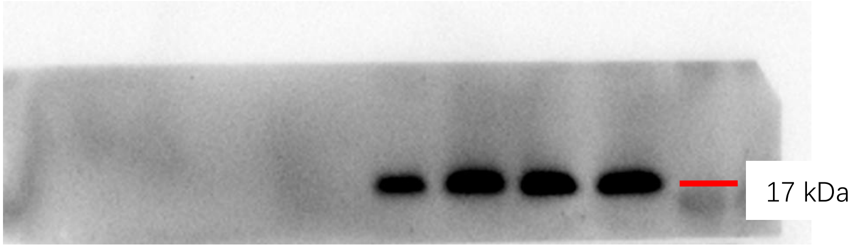

Cleaved Casp1

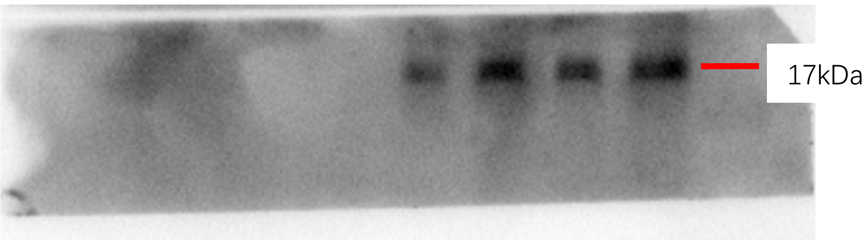

NLRP3

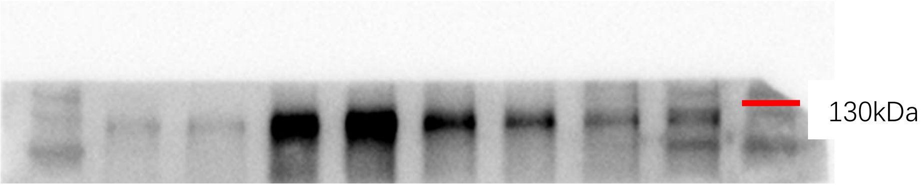

Pro-Casp1

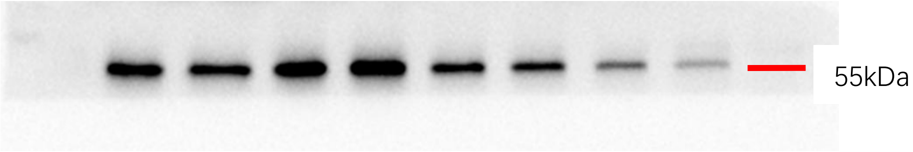

Pro IL-1β

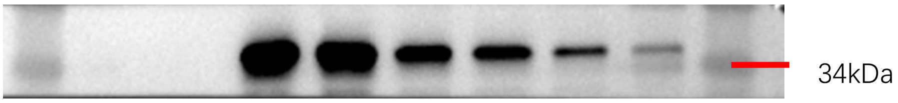

ASC

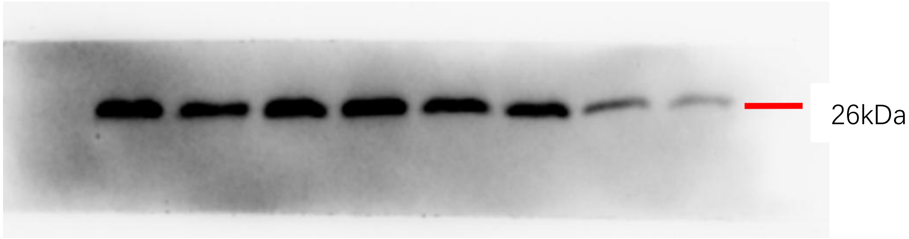

Supplement: Supplementary file 5 — Appendix S1: cns70660‐sup‐0005‐AppendixS1.pdf. [file CNS-31-e70660-s003.pdf]
